# Trans‐generational plasticity in response to immune challenge is constrained by heat stress

**DOI:** 10.1111/eva.12473

**Published:** 2017-04-14

**Authors:** Olivia Roth, Susanne H. Landis

**Affiliations:** ^1^ GEOMAREvolutionary Ecology of Marine FishesHelmholtz Centre for Ocean ResearchKielGermany

**Keywords:** environment, epigenetics, gene expression, global change, immune defence, parasites, parental effects, pipefish

## Abstract

Trans‐generational plasticity (TGP) is the adjustment of phenotypes to changing habitat conditions that persist longer than the individual lifetime. Fitness benefits (adaptive TGP) are expected upon matching parent–offspring environments. In a global change scenario, several performance‐related environmental factors are changing simultaneously. This lowers the predictability of offspring environmental conditions, potentially hampering the benefits of TGP. For the first time, we here explore how the combination of an abiotic and a biotic environmental factor in the parental generation plays out as trans‐generational effect in the offspring. We fully reciprocally exposed the parental generation of the pipefish *Syngnathus typhle* to an immune challenge and elevated temperatures simulating a naturally occurring heatwave. Upon mating and male pregnancy, offspring were kept in ambient or elevated temperature regimes combined with a heat‐killed bacterial epitope treatment. Differential gene expression (immune genes and DNA‐ and histone‐modification genes) suggests that the combined change of an abiotic and a biotic factor in the parental generation had interactive effects on offspring performance, the temperature effect dominated over the immune challenge impact. The benefits of certain parental environmental conditions on offspring performance did not sum up when abiotic and biotic factors were changed simultaneously supporting that available resources that can be allocated to phenotypic trans‐generational effects are limited. Temperature is the master regulator of trans‐generational phenotypic plasticity, which potentially implies a conflict in the allocation of resources towards several environmental factors. This asks for a reassessment of TGP as a short‐term option to buffer environmental variation in the light of climate change.

## 1 Introduction

How species and ecosystems respond to rapid environmental alterations and spatial heterogeneity is crucial for the maintenance of biodiversity, in particular in the light of strong selection imposed by global change (Carroll et al., 2014; Chevin, Lande, & Mace, 2010; Hoffmann & Sgro, 2011). Responses to novel environmental conditions involve phenotypic acclimatization or genetic adaptation (Agrawal, Brodie, & Brown, 2001; Gienapp, Teplitsky, Alho, Mills, & Merila, 2008; Reusch, 2014; Valladares et al., 2014). While the latter is a population‐level process over multiple generations, phenotypic plasticity operates within the lifetime of an individual (Franks & Hoffmann, 2012; Gerken, Eller, Hahn, & Morgan, 2015; Harms et al., 2014; Kellermann, van Heerwardsen, Sgro, & Hoffmann, 2009). Phenotypic plasticity has the ability to speed up the process of adaptive evolution by constituting conditions that later become genetically assimilated (Badyaev 2005; Ghalambor, McKay, Carroll, & Reznick, 2007; Ghalambor et al., 2007; Pigliucci & Murren 2003; Price, Qvarnstrom & Irwin 2003; Robinson & Dukas 1999; Schlichting 2004; West‐Eberhard 2003). In addition, to overcome the lag phase until genetic adaptation takes effect, phenotypic plasticity, that is, the response to environmental conditions, can be passed on from parents to offspring (trans‐generational plasticity, TGP). In TGP, the phenotypic changes in offspring are a response to the parental environment or phenotype, rather than to the offspring environment (Burgess & Marshall, 2014; Mousseau & Fox, 1998). If the parental environment is a good predictor of the offspring environment, TGP can be adaptive and boost the survival of the exceptionally sensitive early developmental stages, with long‐lasting benefits until adulthood (Kirkpatrick & Lande, 1989; Kristensen, 1995; Mousseau & Fox, 1998; Rossiter 1996). This enables a rapid acclimatization to fast changing, fluctuating or spatially heterogeneous environmental conditions. TGP was shown as an efficient short‐time effect buffering impacts of shifts in abiotic factors, such as temperature variations and increased CO_2_ concentrations observed in the ocean during ongoing climate change (e.g., Bonduriansky & Day, 2009; Donelson, Munday, McCormick, & Pitcher, 2012; Shama, Strobel, Mark, & Wegner, 2014). Growth, aerobic scope and mitochondrial respiration were higher when offspring were reared under the conditions matching the parental experience (Donelson et al., 2012; Miller, Watson, Donelson, McCormick, & Munday, 2012; Salinas & Munch, 2012; Shama et al., 2014). TGP influences the phenotype of the offspring (Badyaev & Uller, 2009; Bonduriansky & Day, 2009; Wolf & Wade, 2009) without any change in the nucleotide sequence of the DNA (Bonduriansky & Day, 2009; Kirkpatrick & Lande, 1989). In addition to life‐history adjustments (e.g., enhanced growth and survival), TGP can impact epigenetic modifications via DNA methylation and histone acetylation that alter gene expression profiles (Ho & Burggren, 2010; Holeski, Jander, & Agrawal, 2012; Ragunathan, Jih, & Moazed, 2015; Rassoulzadegan et al., 2006).

Under ongoing global change, shifts in abiotic factors (such as increased temperature variations or higher CO_2_ levels) are accompanied by changes in biotic factors (e.g., parasites, predators) that may also favour acclimatization via the transfer of parental environmental experience (Vidal‐Martinez, Pech, Sures, Purucker, & Poulin, 2010). Parasites are abundant and affect all living organisms (Price, 1990; Windsor, 1998), but their distribution, replication and virulence are susceptible to the conditions in their respective environments (Lafferty & Kuris, 1999; Marcogliese, 2008). The coevolutionary arms race between host and parasite (Anderson & May, 1982; Hamilton, 1980) selects for host immune systems (Altizer, Harvell, & Friedle, 2003; Boots & Bowers, 2004) that exhibit genetic specificity and phenotypic plasticity enabling them to combat abundant parasites and pathogens (Schmid‐Hempel, 2011). If environmental change induces a shift in the parasite assemblage, this host plasticity promotes an efficient response that mediates acclimatization (Lazzaro & Little, 2009). The plasticity can be transferred from the parents to the offspring in invertebrates and vertebrates (trans‐generational immune priming, TGIP; Beemelmanns & Roth, 2016a, 2016b, 2017; Grindstaff, Brodie, & Ketterson, 2003; Hasselquist & Nilsson, 2009; Roth, Klein, Beemelmanns, Scharsack, & Reusch, 2012; Sadd, Kleinlogel, Schmid‐Hempel, & Schmid‐Hempel, 2005). TGIP boosts offspring immunity and is in vertebrates of particular importance during early life stages, when mortality selection is high due to the immature adaptive immune system (Hasselquist & Nilsson, 2009). As a particular case of trans‐generational phenotypic plasticity, TGIP has the ability to compensate for the impact of alterations in pathogen and parasite assemblies as predicted under climate change.

Trans‐generational plasticity has been shown to buffer single abiotic and biotic changes (Beemelmanns & Roth, 2016b; Donelson et al., 2012; Roth, Klein, et al., 2012; Salinas & Munch, 2012; Shama et al., 2014). However, whether TGP also has the potential to compensate multiple environmental modifications simultaneously remains unknown. Addressing this gap is of particular importance as anthropogenically induced global change alters a wealth of environmental factors concurrently, major threads in the ocean are higher temperature variations, enhanced CO_2_ concentrations, a drop in salinity and the intermingled increased pathogen replication and virulence (Brook, Sodhi, & Bradshaw, 2008; Hoegh‐Guldberg et al., 2007; Walther et al., 2002). Trans‐generational acclimatization towards different environmental variables may involve the same physiological mechanisms. In such a scenario, TGP as a response to two changing environmental factors could be more than additive resulting in a synergistic beneficial offspring response. However, both TGP and TGIP are costly (Sadd & Schmid‐Hempel, 2009; Sheldon & Verhulst, 1996), and they may only be adaptive when the offspring environment is predictable and matches the parental environment (Burgess & Marshall, 2014; Donald‐Matasci, 2013; Fischer, Taborsky, & Kokko, 2011; Marshall & Uller, 2007; Mousseau & Fox, 1998). For example, if a temperature shift in the parental generation is combined with induced pathogen prevalence, the energetic costs for plastic acclimatization can rise. In addition, the probability for matching parental and offspring environments is diminished. This increases the selection for genetic adaptation, but also implies greater costs on the parental and offspring side due to reduced reproduction and survival (Carroll et al., 2014). This may lower the extent and benefits of TGP and induces costs. Two environmental factors that change in the parental generation could thus have antagonistic effects on offspring fitness, fading out the impact of TGP.

Investigating the synergistic and antagonistic impact of two changing environmental factors in the parental generation can help to unravel the limits of TGP.

To address the trans‐generational impact of two interacting parental abiotic and biotic environmental changes, the parental generation of wild‐caught pipefish of the species *Syngnathus typhle* were in a fully reciprocal mating design exposed to an ambient and an elevated temperature regime (18°C [cold] vs. 23°C [hot]) and immunological challenges with heat‐killed *Vibrio* bacteria or no immunological activation. 18°C represents the ambient temperature during the breeding season along the coast of the western Baltic Sea, while increasing temperatures from 18°C to 23°C within 7 days represent a heatwave, which both in the temperature and the rate of change resemble natural conditions that have been observed in coastal environments during the last 20 years (Benston, Stephenson, Christensen, & Ferro, 2007; Schär & Jendritzky, 2004; Team, 2008; Vidale, 2007). The exposure to an immune challenge with heat‐killed *Vibrio* bacteria mimicked an immunological activation upon a successful parasite infection. Pipefish pairs were fully reciprocally formed and mated (four parental environments: Vibrio cold: VC; Vibrio hot: VH; naïve cold: NC, naïve hot: NH). Duration of male pregnancy and clutch size were evaluated. To simulate matching and nonmatching environmental conditions, offspring of each family were split directly after birth into cold or hot offspring environment. Half of the offspring from each temperature treatment were then exposed to an immune challenge with heat‐killed *Vibrio* bacteria (four offspring environments: VC; VH; NC, NH), upon which gene expression and life‐history responses were assessed.

We hypothesize that parental immune challenge and heatwave share the same physiological mechanisms and thus have an interactive effect on offspring performance. The direction of these effects can either be antagonistic such that the impact of parental *Vibrio* exposure on offspring immune defence (TGIP) is reduced when heat stress is experienced simultaneously. Or the experience of two environmental factors in the parental generation can also have synergistic beneficial impact on offspring performance, in particular in case of a matching parental and offspring environment (Burgess & Marshall, 2014; Mousseau & Fox, 1998; Salinas & Munch, 2012; Uller, 2008).

Alternatively, we hypothesize that parental immune challenge and temperature change do not interact but involve separate physiological mechanisms relying on segregated resource pools. Focusing on these two hypotheses, we assessed expression of a limited gene set (44) involved in immune defence or DNA modification and histone modification with a known role in TGIP (Beemelmanns & Roth, 2016a, 2016b; Roth, Klein, et al., 2012). As life‐history responses, we measured duration of male pregnancy, clutch size and offspring size.

Consistent with earlier studies in the pipefish *S. typhle,* parental *Vibrio* exposure induced offspring gene expression. However, if parents were exposed to elevated temperatures (23°C [hot]) in combination with an exposure to *Vibrio*, the impact of TGIP almost disappeared. This supports the hypothesized interactive effects of parental immune challenge and exposure to elevated temperature on offspring gene expression.

## 2 Material and methods

### 2.1 Experiment

The parental pipefish generation was caught end of April 2014 by snorkelling with handnets in a seagrass meadow in northern Germany near Gelting in 1–3 m water depth. Fish were transported within 2 hr after capture to our aquaria facilities at GEOMAR in Kiel and slowly acclimated to laboratory conditions (18°C, salinity according to natural conditions in Kiel harbour [14–18 PSU]). During this time, animals were kept sex‐separated in six 200‐L barrels. All animals were healthy and did not show any symptoms of ongoing infections. On May 16, fish were moved into the glass aquaria system, two fish of the same sex per 80‐L aquarium, a total of 64 fish in 32 aquaria. The next day, these aquaria were randomly assigned to either the “cold” or the “hot” circulation system (16 aquaria each). In the aquaria belonging to the “hot” parental group, temperature was slowly raised by 1°C per day until 23°C was reached. This represents a recent heatwave scenario that can occur in summer in coastal waters of the Baltic Sea (Benston et al., 2007; Schär & Jendritzky, 2004). The other aquaria remained at 18°C. Fresh Baltic sea water was added daily (300 L/day, about 10% of the water in the aquaria system). In addition, water was exchanged between the two circulation systems to avoid confounding effects of the distinct aquaria systems.

On May 22, animals from the cold and the hot group were either exposed to an immune challenge with heat‐killed *Vibrio* bacteria (peritoneal injection of 50 μl of 10^9^ bacteria/ml) of an Italian *Vibrio* isolate I9K1 (Beemelmanns & Roth, 2016a, 2016b; Roth, Keller, Landis, Salzburger, & Reusch, 2012; Roth, Klein, et al., 2012), or they were left naïve. In each aquarium, one female and one male were placed that were either both immune‐challenged but kept at cold temperatures (VC), both immune‐challenged but exposed to elevated temperatures (VH), both not immune‐challenged and kept at cold temperatures (NC) or both not immune‐challenged and exposed to elevated temperatures (NH). These pairs were designated as parental generation according to the four different treatments: NC, NH, VC, VH, eight replicates each, in total 32 pairs. A total of 29 pairs mated within 48 hr (7 NC, 6 NH, 8 VC, 8 VH), thereafter named families. The temperature was kept at 18°C or 23°C, respectively, throughout pregnancy. During pregnancy, seven families were either lost due to death (two families), as they jumped out of the aquaria (three families), or as they lost their brood (two families). Males of 22 families successfully gave birth to offspring (seven NC, five NH, six VC, four VH) between June 9 and 18. Pregnancy lasted on average 17.4 ± 0.167 days (mean ± *SE*) for animals kept at hot temperatures, and 23.6 ± 0.55 days for animals kept at cold temperatures. Males were checked for signs of ongoing birth four times a day. Immediately after birth, the clutch was split and transferred into small aquaria (2 L), one kept at 18°C, the other one at 23°C. The small aquaria were swimming in the large tanks of the aquaria system due to their polystyrene surrounding, and water exchange was permitted over two circular sections that were cut and covered with fine‐mesh nets. Eight days after birth, juveniles of each half‐clutch were again split and either exposed to an immune challenge with heat‐killed *Vibrio* bacteria over a pricking with a needle dipped in a solution of 10^10^ heat‐killed *Vibrio* bacteria/ml, or stayed without an immunological treatment (naïve) as described in Beemelmanns and Roth (2016a, 2016b). For this treatment, four families per parental treatment (16 families in total) with an equal distribution of five replicates per each of the four offspring treatment groups (NC, NH, VC, VH) were used (20 offspring per family resulting in 16 × 20 = 320 animals). 24 hr after the offspring immune challenge, the total length of the animals was measured in mm (from the tip of the snout to the tip of the caudal fin), and animals were killed by decapitation and for later usage stored in RNA later at −20°C.

We quantified the mRNA level of 44 preselected target genes already used in previous studies (Beemelmanns & Roth, 2016a; Birrer, Reusch, & Roth, 2012; Roth, Keller, et al., 2012; Roth, Klein, et al., 2012) with quantitative real‐time polymerase chain reaction (qPCR). The genes were originally identified and selected from transcriptomes of several *S. typhle* individuals that were previously exposed to natural *Vibrio* isolates (Haase et al. 2013). Total RNA was extracted of 320 whole‐body samples with an RNeasy96 Universal‐Tissue Kit (Qiagen, Venlo, the Netherlands) according to the manufacturer's protocol. Extraction yields were measured by a spectrophotometer (NanoDrop ND‐1000; Peqlab, Erlangen, Germany) to allow a reverse transcription into cDNA via a QuantiTect Reverse Transcription Kit (Qiagen) of a fixed amount of 800 ng/μl. The expression of 48 genes was measured simultaneously for all samples using a BioMark™ HD system (Fluidigm, South San Francisco, CA, USA) based on 96.96 dynamic arrays (GE chips). A pre‐amplification step was performed by mixing a 500 nM primer pool of all 48 primers with 2.5 ml TaqMan PreAmp Master Mix (Applied Biosystems, Waltham, MA, USA), and 1.25 μl cDNA per sample. The mixture was pre‐amplified (10 min at 95°C; 14 cycles: 15 s at 95°C, 4 min at 60°C), and the PCR products were diluted 1:10 with low EDTA‐TE buffer. For chip loading, a sample mix was prepared by combining 3.5 ml 2× Ssofast‐EvaGreen Supermix with Low ROX (Bio‐Rad Laboratories, Hercules, CA, USA) with 0.35 μl 20 × DNA Binding Dye Sample & Assay Loading Reagent (Fluidigm) and 3.15 μl pre‐amplified PCR products. An assay mix was prepared by combining 0.7 μl of 50 μM primer pair mix, 3,5 μl Assay Loading Reagent (Fluidigm) and 3.15 μl low EDTA‐TE buffer. At the end, 5 μl of each sample and assay mix were filled into the GE chips and measured in the BioMark system, applying the GE‐fast 96.96 PCR protocol according to the manufacturer's instructions (Fluidigm). The samples were distributed randomly across chips, and each of these included no template controls, controls for gDNA contamination (−RT) and standards and two technical replicates per sample and gene (protocol and reference genes according to Beemelmanns and Roth (2016a, 2016b)).

For each of the two technical replicates per sample, the mean cycle time (Ct), the standard deviation (*SD*) and the coefficient of variance (CV) were calculated. If CV was larger than 4%, samples were removed due to potential measurement errors (Bookout and Mangelsdorf 2003). The housekeeping genes ubiquitin (*Ubi*) and ribosome protein (*Ribop*) showed the highest stability (geNorm M > 0.85) (Hellemans et al. 2007). Their geometric mean was thus used to quantify relative expression of each target gene by calculating ∆Ct values. A total of 27 animals had to be excluded from further statistical analyses due to measurement errors in gene expression profiles.

### 2.2 Data analysis and statistics

This study aimed to evaluate how the combination of parental immune challenge and temperature change affected duration of male pregnancy and clutch size. Upon offspring exposure to both parental temperature treatment and *Vibrio* immune challenge (split design within each family) expression of immune genes, genes mediating epigenetic signalling (DNA modification and histone modification) and impact on offspring body size, an important life‐history trait, were evaluated. This permitted the study of usage of similar or distinct physiological mechanism when two environmental stressors were applied both during the parental and the offspring generation. Doing so, we could assess the potentially interactive effect of TGP according to two environmental alterations, and its adaptive characteristics in case of matching or nonmatching parental and offspring environmental conditions.

The data analysis was performed in R (v3.2.2) according to Beemelmanns and Roth (2016a, 2016b) with minor modifications. A permutational multivariate analysis of variance (PERMANOVA) was applied for immune gene expression (29 target genes) as well as epigenetic regulation genes (15 target genes) (320 samples).

The PERMANOVA model (“vegan” package—”adonis” function in R) was based on a Bray–Curtis matrix of nontransformed ∆Ct values with 1,000 permutations per model. Our data fulfilled the requirements for multivariate homogeneity of group dispersion, which was tested with betadisper (“vegan” package). We applied tparent (temperature parents), Vparent (Vibrio exposure parents), toffspring (temperature offspring), Voffspring (Vibrio exposure offspring) as fixed factors and included “family” as random effect (strata = family). The PERMANOVA that included all genes as response variables was followed by PERMANOVAs for the following functional gene groups: (i) adaptive immune response, (ii) innate immune response, (iii) complement system, (iv) methylation/demethylation and (v) acetylation/deacetylation) (all genes in Table 1).

**Table 1:** All genes assessed and discussed in this manuscript, grouped according to their functional categories. Gene names and functions are given for each gene. In the references, the according primers and accession numbers on NCBI can be found

Gene	Category	Gene name	Function	Reference & primer sequence
*Lymphag 75*	Adaptive	Lymphocyte antigen 75	Antigen recognition	Birrer et al. (2012)
*HIVEP 2*	Adaptive	Human immunodeficiency virus type I enhancer 2	VDJ recombination, MHC binding	Beemelmanns and Roth (2016a)
*HIVEP 3*	Adaptive	Human immunodeficiency virus type I enhancer 3	VDJ recombination, MHC binding	Beemelmanns and Roth (2016a)
*CD45*	Adaptive	CD45 (leucocyte common antigen)	T‐ and B‐cell antigen receptor signalling	Beemelmanns and Roth (2016a)
*Integrin*	Adaptive	Integrin‐beta 1	Adhesion of immunoglobulins	Beemelmanns and Roth (2016a)
*IgM*	Adaptive	Immunoglobulin light chain	Antigen/pathogen recognition	Beemelmanns and Roth (2016a)
*TAP*	Adaptive	Tap‐binding protein (tapasin)	Antigenic peptide transport & loading	Beemelmanns and Roth (2016a)
*Bcell.rap*	Adaptive	B‐cell receptor‐associated protein	T‐ and B‐cell regulation activity	Roth, Klein, et al. (2012)
*Lympcyt*	Adaptive	Lymphocyte cytosolic protein 2	T‐cell development and activation	Beemelmanns and Roth (2016a)^2014^
*LectpI*	Innate	Lectin protein type I	Pathogen recognition receptor	Beemelmanns and Roth (2016a)
*LectpI*	Innate	Lectin protein type II	Pathogen recognition receptor	Beemelmanns and Roth (2016a)
*cf*	Innate	Coagulation factor II	Blood clotting and inflammation	Birrer et al. (2012)
*Hsp60*	Innate	Heat‐shock protein 60	Chaperone, general stress response	Roth, Klein, et al. (2012)
*Ik.cyto*	Innate	IK cytokine	Inhibits interferon gamma	Beemelmanns and Roth (2016a)
*IL10*	Innate	Interleukin‐10	Regulation of macrophage activity	Birrer et al. (2012)
*kin*	Innate	Kinesin	Intracellular transport	Roth, Klein, et al. (2012)
*nramp*	Innate	Natural resistance‐associated macrophage protein	Macrophage activation	Roth, Klein, et al. (2012)
*TSPO*	Innate	Translocator protein	Inflammatory response	Roth, Klein, et al. (2012)
*LPS:TNF*	Innate	LPS‐induced TNF‐alpha factor (LITAF)	Cytokine expression	Beemelmanns and Roth (2016a)
*calcrul*	Innate	Calreticulin	Phagocytosis promotion	Beemelmanns and Roth (2016a)
*intf*	Innate	Interferon‐induced transmembrane protein 3	Viral entry into host cell, antiviral	Beemelmanns and Roth (2016a)
*IL8*	Innate	Interleukin‐8	Phagocytosis, inflammation	Beemelmanns and Roth (2016a)
*Tyroprot*	Innate	Tyroproteinkinase	Cytokine receptor signalling	Beemelmanns and Roth (2016a)
*ck7*	Innate	Chemokine 7	Chemotaxis for immune cells	Beemelmanns and Roth (2016a)
*AIF*	Innate	Allograft inflammation factor	Inflammatory response, allograft rec	Roth, Klein, et al. (2012)
*transferin*	Innate	Transferrin	Bacterial growth prevention	Beemelmanns and Roth (2016a)
*C1*	Complement	Recognition subcomponent (C1q)	Antigen–antibody complex formation	Beemelmanns and Roth (2016a)
*C3*	Complement	Complement component 3	Activation of complement system	Birrer et al. (2012)
*C9*	Complement	Complement component 9	Membrane attack complex, lysis	Roth, Klein, et al. (2012)
*JmjcPhD*	Methylation	Lysine‐specific demethylase 5B	Histone demethylation	Beemelmanns and Roth (2016a)
*No66*	Methylation	Lysine‐specific‐histone demethylase No66	Histone demethylation	Beemelmanns and Roth (2016a)
*TPR*	Methylation	Lysine‐specific demethylase 6A	Histone demethylation	Beemelmanns and Roth (2016a)
*DnMt1*	Methylation	DNA‐Methyltransferase 1	Maintenance methylation	Beemelmanns and Roth (2016a)
*DnMt3a*	Methylation	DNA‐Methyltransferase 3a	*De novo* methylation	Beemelmanns and Roth (2016a)
*DnMt3b*	Methylation	DNA‐Methyltransferase 3b	*De novo* methylation	Beemelmanns and Roth (2016a)
*N6admet*	Methylation	N(6)‐adenine‐specific DNA‐Methyltransferase	DNA‐methyltransferase	Beemelmanns and Roth (2016a)
*ASH*	Methylation	Histone methyltransferase	Histone methyltransferase	Beemelmanns and Roth (2016a)
*HDAC1*	Acetylation	Histone deacetylase 1‐like	Histone deacetylation	Beemelmanns and Roth (2016a)
*HDAC3*	Acetylation	Histone deacetylase 3‐like	Histone deacetylation	Beemelmanns and Roth (2016a)
*HDAC6*	Acetylation	Histone deacetylase 6‐like	Histone deacetylation	Beemelmanns and Roth (2016a)
*Hemk2*	Acetylation	HemK‐methyltransferase family member 2	Histone deacetylation	Beemelmanns and Roth (2016a)
*MYST*	Acetylation	Histone acetyltransferase	Histone acetylation	Beemelmanns and Roth (2016a)
*BROMO*	Acetylation	Histone acetyltransferase	Histone acetylation	Beemelmanns and Roth (2016a)

Statistical univariate approaches were applied for body size and as post hoc tests for factors showing a significant effect in the PERMANOVAs to evaluate the contribution of single genes. Only factors with a significant effect in the PERMANOVAs were considered. A linear mixed effect model (“nmle” package—”lmer” function in R) was fitted using tparent (temperature parents), Vparent (*Vibrio* exposure parents), toffspring (temperature offspring), Voffspring (Vibrio exposure offspring) as fixed factors and “family” as random effect. Prior to the analysis, response variables (gene expression, size) were box‐cox‐transformed, and data and residuals were tested for normal distribution and variance homogeneity (Shapiro–Wilk test, Levene's test). Post hoc Student's *t* tests to examine interactions in more detail followed significant ANOVAs.

The impact of the parental immunological treatment (Vparent) and parental temperature treatment (tparent) on duration of pregnancy and clutch size was analysed in an ANOVA with Vparent and tparent as fixed factors.

**Figure 1:**
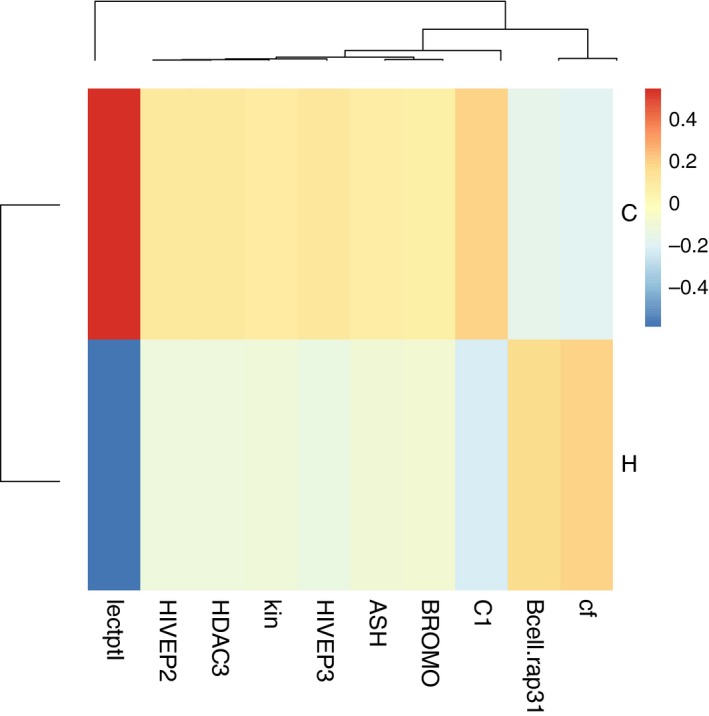
A heatmap showing sample means per parental treatment of all genes that were significantly affected by the tparent main effect (*n* = 10), normalized by the overall mean of the gene (−∆∆*C*_t_), displayed for either parental ambient (cold: C) or elevated temperature (hot: H) environment

**Figure 2:**
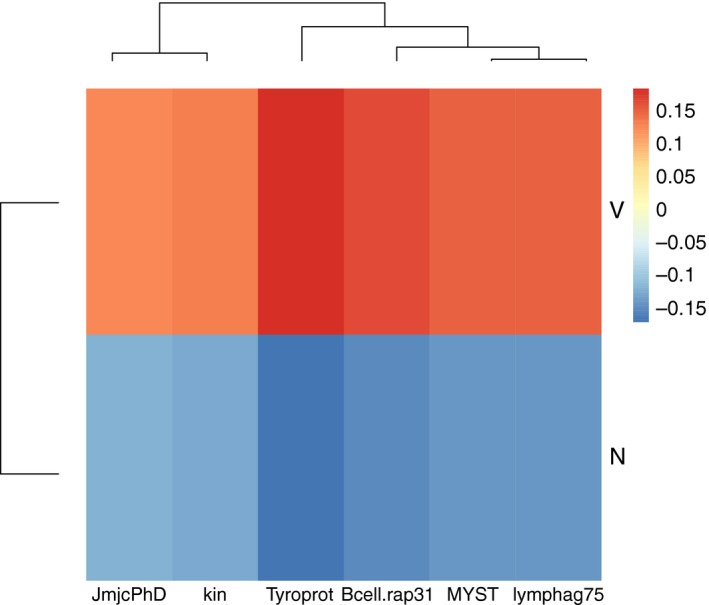
A heatmap showing sample means per parental treatment of all genes that were significantly affected by the Vparent main effect (*n* = 6), normalized by the overall mean of the gene (−∆∆*C*_t_), displayed for either parental Vibrio immune challenge (Vibrio: V) or control (naïve: N)

**Figure 3:**
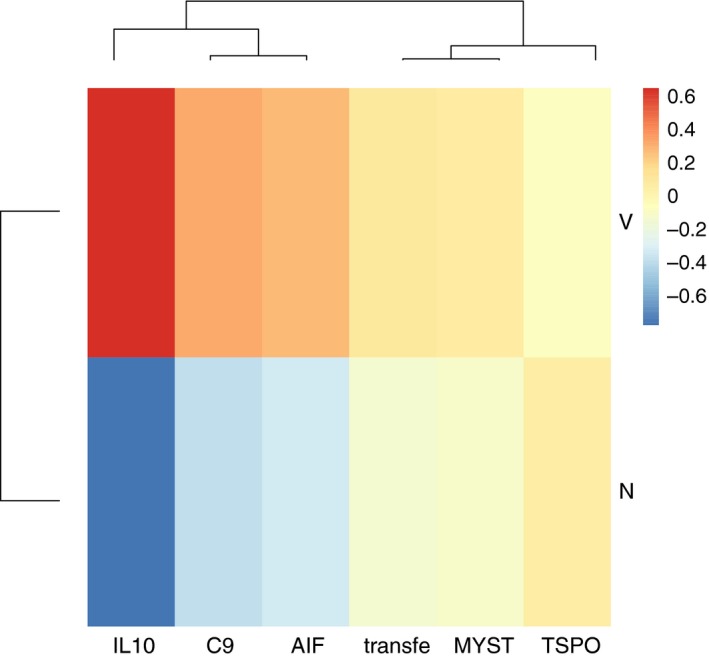
A heatmap showing sample means per offspring treatment of all genes that were significantly affected by the Voffspring main effect (*n* = 6), normalized by the overall mean of the gene (−∆∆*C*_t_), displayed for either offspring *Vibrio* immune challenge (Vibrio: V) or control (naïve: N)

**Figure 4:**
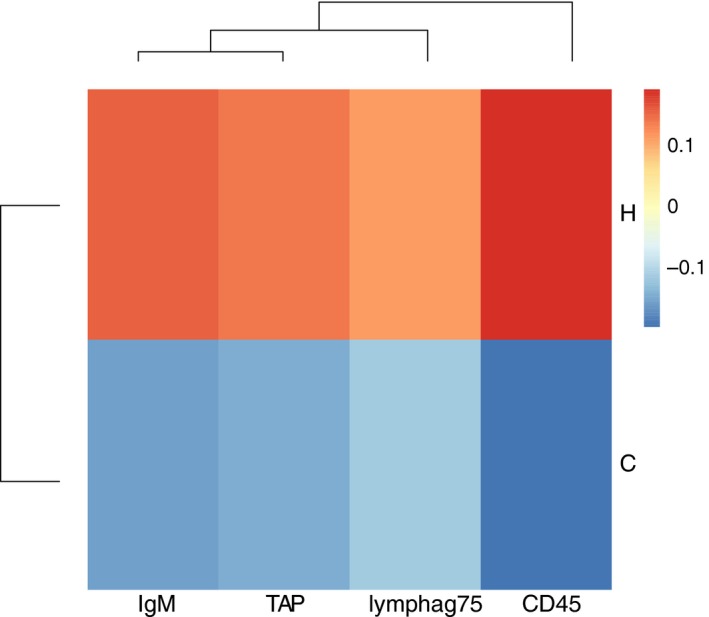
A heatmap showing sample means per offspring treatment of all genes that were significantly affected by the toffspring main effect (*n* = 4), normalized by the overall mean of the gene (−∆∆*C*_t_), displayed for either offspring ambient (cold: C) or elevated temperature (hot: H) environment

**Figure 5:**
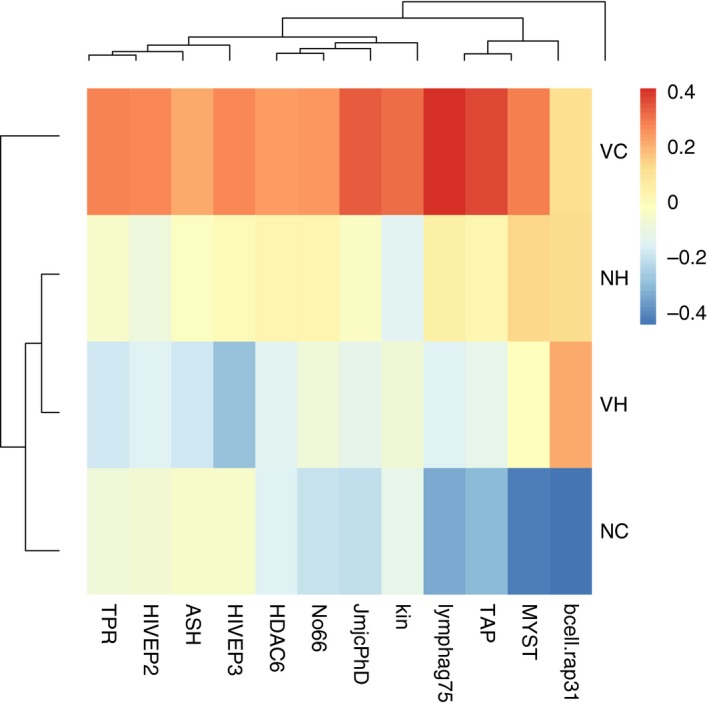
A heatmap showing sample means per parental treatment of all genes that were significantly affected by the Vparent × tparent interaction (*n* = 12), normalized by the overall mean of the gene (−∆∆*C*_t_), displayed for either parental Vibrio cold (VC), Vibrio hot (VH), naïve cold (NC) or naïve hot (NH) parental environment

**Figure 6:**
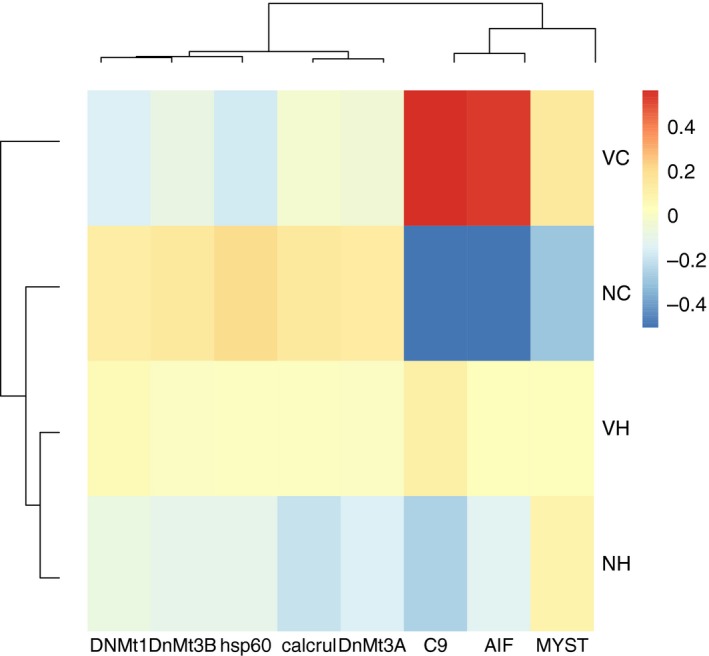
A heatmap showing sample means per offspring treatment of all genes that were significantly affected by the Voffspring × toffspring interaction (*n* = 8), normalized by the overall mean of the gene (−∆∆*C*_t_), displayed for either parental Vibrio cold (VC), Vibrio hot (VH), naïve cold (NC) or naïve hot (NH) offspring environment

**Figure 7:**
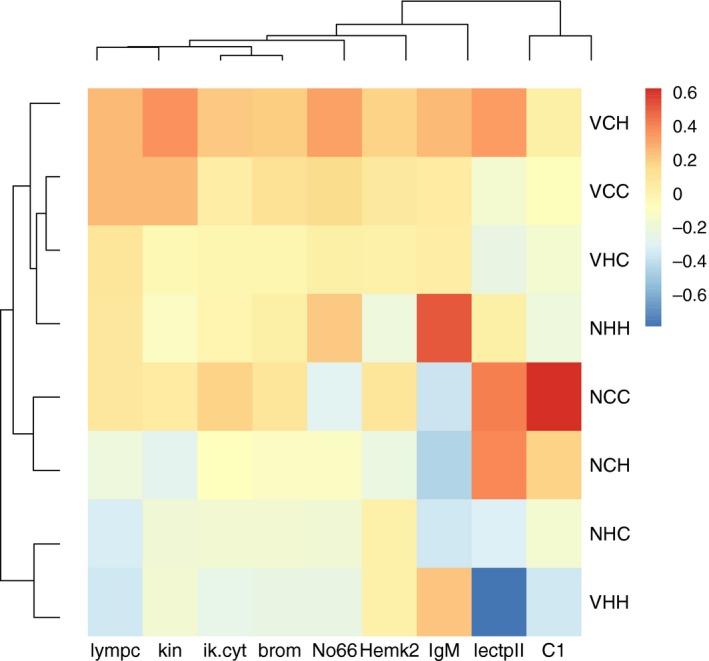
A heatmap showing sample means per parental × offspring treatment of all genes that were significantly affected by the tparent × Vparent × toffspring interaction (*n* = 9), normalized by the overall mean of the gene (−∆∆*C*_t_), displayed for either parental Vibrio cold offspring cold (VCC), parental Vibrio hot offspring cold (VHC), parental Vibrio cold offspring hot (VCH), parental Vibrio hot offspring hot (VHH), parental naïve cold offspring cold (NCC), parental naïve hot offspring cold (NHC), parental naïve cold offspring hot (NCH) or parental naïve hot offspring hot (NHH)

Heatmaps were depicted for graphical visualization of gene expression (Figures 1, 2, 3, 4, 5, 6, 7). For normalization (–∆∆*C*_t_), the ∆Ct value of each sample was subtracted from the average ∆*C*_t_ value. Means of –∆∆*C*_t_ values of either the significant main effects or the interactions are shown.

## 3 Results

### 3.1 Gene expression

#### 3.1.1 Multivariate analysis—all genes

Both parental temperature and bacterial experience, but also offspring bacterial exposure (tparent, Vparent and Voffspring), significantly changed gene expression in offspring (PERMANOVA over all genes). In addition, the interaction of the two parental environmental factors, the interaction of the offspring temperature and bacterial exposure (tparent × Vparent, toffspring × Voffspring), and the interaction of the two parental factors with the temperature offspring experienced (tparent × Vparent × toffspring) influenced offspring gene expression profiles (Table 2, all genes). This supports our hypothesis that two interacting environmental changes (Vparent × tparent interaction) in the parental generation result in a different offspring gene expression profile than a single environmental change. The effects identified, when two parental environmental changes were applied, were not additive. Matching environmental parental and offspring conditions resulted for several genes in a specific differential expression implying TGP (significant tparent × Vparent × toffspring interaction). However, its adaptive characteristics could mostly not be confirmed (missing Vparent × Voffspring and tparent × toffspring interactions, and no Vparent × tparent × Voffspring × toffspring interaction).

**Table 2:** PERMANOVAs for all genes (44 genes) or the functional categories of genes: innate immune genes (innate imm [17]), adaptive immune genes (adaptive imm [9]), complement system genes (complement [3]), methylation genes (methylation [8]) and acetylation genes (acetylation [6]). Tparent (tpar), Vparent (Vpar), toffspring (toff) and Voffspring (Voff) were included as fixed factors with all interactions. Strata = family was used to account for the random family effect. Significant effects (*p* < 0.05) are indicated with asterisks * and in bold italic letters. R code: Adonis (dist.all~tparent*Vparent*toffspring*Voffspring, method = “Euclidean,” permutations = 1,000)

PERMANOVAs factors	*df*	All genes (44)		Innate imm (17)		Adaptive imm (9)		Complement (3)		Methylation (8)		Acetylation (6)	
		*F* mod	Pr (>*F*)	*F* mod	Pr (>*F*)	*F* mod	Pr (>*F*)	*F* mod	Pr (>*F*)	*F* mod	Pr (>*F*)	*F* mod	Pr (>*F*)
**tpar**	1	7.954	<.001*	9.162	<.001*	3.947	.005*	14.789	<.001*	4.556	.003*	5.751	.003*
**Vpar**	1	5.971	<.001*	5.078	.003*	8.303	<.001*	5.343	.008*	3.890	.006*	5.685	.002*
toff	1	1.397	.180	0.526	.785	5.136	.003*	2.268	.098	1.511	.180	1.337	.225
**Voff**	1	5.710	<.001*	2.954	.017*	0.571	.729	9.698	<.001*	0.992	.406	1.696	.155
**tpar:Vpar**	1	6.876	<.001*	7.132	<.001*	7.666	<.001*	10.115	<.001*	7.492	<.001*	15.53	<.001*
tpar:toff	1	0.008	.517	0.778	.543	1.178	.290	1.957	.123	0.281	.930	0.810	.477
Vpar:toff	1	1.447	.144	1.912	.086	0.996	.401	0.774	.423	2.077	.065	2.139	.087
tpar:Voff	1	0.5401	.867	0.682	.645	0.523	.763	0.412	.703	1.166	.281	0.130	.965
Vpar:Voff	1	1.044	.378	1.706	.120	1.004	.406	0.387	.674	0.788	.486	0.450	.804
**toff:Voff**	1	1.930	.039*	1.457	.186	0.983	.397	3.024	.039*	3.137	.013*	5.114	.002*
**tpar:Vpar:toff**	1	2.458	.011*	1.466	.183	2.578	.034*	0.746	.463	2.103	.092	3.636	.018*
tpar:Vpar:Voff	1	0.703	.705	0.626	.682	1.400	.195	0.691	.476	1.885	.104	0.374	.790
tpar:toff:Voff	1	1.023	.398	1.231	.279	1.146	.304	1.689	.195	0.418	.825	0.441	.741
Vpar:toff:Voff	1	0.756	.655	0.465	.815	1.585	.154	0.352	.727	0.991	.369	1.507	.120
tpar:Vpar:toff:Voff	1	1.054	.363	1.608	.127	0.482	.778	1.388	.261	1.091	.336	0.628	.602
Residuals/Rsquare	276	0.874		0.882		0.880		0.837		0.895		0.895	
Total/Rsquare	291	1.000		1.000		1.000		1.000		1.000		1.000	

#### 3.1.2 Multivariate analysis—functional gene groups

All functional groups of genes showed similar patterns (PERMANOVAs for genes involved in (i) adaptive immune response, (ii) innate immune response, (iii) complement system, (iv) methylation/demethylation and (v) acetylation/deacetylation). In all functional groups, significant effects were attributed to the parental temperature and bacterial environment (tparent and Vparent), and their interaction (tparent × Vparent). The bacterial offspring exposure (Voffspring) changed the expression of innate immune genes and of genes from the complement system. The expression of genes involved in DNA modification and histone modification (methylation/demethylation and acetylation/deacetylation) was impacted by the combination of the offspring temperature and bacterial exposure (toffspring × Voffspring), which was also found for genes involved in the complement system. Expression of genes of the adaptive immune system was affected by a combination of both the parental temperature experience and the bacterial experience, and this effect in turn depended on offspring environmental temperature (tparent × Vparent × toffspring). This three‐way interaction effect was also identified for genes mediating acetylation/deacetylation. The temperature offspring were exposed to (toffspring), only influenced expression of adaptive immune genes (Table 2).

#### 3.1.3 Univariate analyses—single genes

For the significant main effects and interactions identified in the PERMANOVA per functional gene group, statistical univariate models (mixed linear effect models) were calculated, followed by post hoc analyses (Student's *t* test) (Tables S1–S5). For ten genes, the parental temperature experience played a significant role (tparent). Of those ten genes, three are involved in innate immune defence (*LectpI, Cf, Kin*), three in adaptive immune defence (*HIVEP2, HIVEP3, Bcell.rap31*) and one in the complement system (*C1)*,* ASH* mediates histone methylation, and *HDAC3* and *BROMO* are involved in histone deacetylation and acetylation. For eight of these ten genes, expression was lower if parents were exposed to an elevated temperature. Only *Cf* and *Bcell.rap31* showed the opposite pattern (Figure 1).

For six genes, parental *Vibrio* injection resulted in a higher expression: *Kin*,* Tyroprot* (innate immune system), *Lymphag75* und *Bcell.rap31* (adaptive immune system), *JmjcPhD* (demethylation of histones) and *MYST* (acetylation of histones) (Figure 2).

Bacterial exposure of offspring (Voffspring) significantly influenced *IL10*,* AIF*,* TSPO*,* transferrin* (innate immune system), as well as *C9* (complement system) and *MYST* (acetylation of histones). *Vibrio* injection of offspring enhanced expression of most genes; only *TSPO* was downregulated (Figure 3).

Experience of elevated temperatures during offspring development induced expression of *lymphag75*,* CD45*,* IgM* and *TAP* (all adaptive immune system) (Figure 4).

The interaction of the two parental environmental conditions (temperature and *Vibrio* injection, tparent × Vparent) affected expression of *Kin* (innate immune system), *Lymphag75, Hivep2, Hivep3, TAP, Bcell.rap31* (all adaptive immune system), *ASH, JmjcPhD, No66* and *TPR* (all methylation/demethylation of histones), *HDAC6* and *MYST* (acetylation/deacetylation of histones) (Figure 5). The offspring descending from the parents that were exposed to *Vibrio* but kept at cold temperatures (VC) showed in eleven of the twelve genes a significantly higher expression than all other groups (NH, VH, NC). Only *Bcell.rap31* was also induced by a temperature increase (NH) or by a combination of a temperature shift and an immune challenge (VH). If parents were kept at ambient water temperature and had no immune challenge, its expression was lowest in the offspring (NC). The expression of *HIVEP2, Kin, JmjcPhD, TPR* and *ASH* was highest in case of a parental Vibrio exposure, but remained unaffected by parental temperature exposure or by the combination of a temperature shift and an immune challenge (VC > NC, NH, VH). In three genes (*TAP, No66, HDAC6*), also an elevation of temperatures during the parental generation and the combination of temperature shift and immune challenge induced the expression, however, to a much smaller extent than the sole parental *Vibrio* immune challenge (VC > NH, VH > NC). Only in one gene (*Lymphag 75*), a difference between the sole temperature shift during the parental generation and the parental immune challenge in combination with a temperature shift was identified (VC > NH > VH > NC). All but the last gene discussed speak for a dominant parental temperature effect, as the expression of the combined parental treatments was always on the same level as if only a temperature change was applied (VH = NH), and always lower than if a sole parental immune challenge was applied (Figure 1). This suggests that TGIP initiated by a *Vibrio* exposure of the parents was only effective at cold temperatures. Hot temperatures hampered the effect of parental *Vibrio* exposure, and no TGIP could be identified in a scenario with two interacting parental challenges (*Vibrio* and temperature) (Figure 5).

*Hsp60, AIF, Calcrul* (all innate immune system), *C9* (complement system), *DnMT1, DnMt3a, DnMt3B* (all DNA methylation) and *MYST* (acetylation of histones) were affected by the interaction of the abiotic and biotic environmental conditions employed during offspring development (toffspring × Voffspring). The pattern found here was not consistent across the different genes. The expression of *Hsp60* and *DnMt1* was lowered if offspring were exposed to elevated temperatures or a *Vibrio* challenge, but not if both factors were changed simultaneously (NC, VH > NH, VC). *DnMT3a* and *DnMT3b* showed a lowered expression, independent of whether offspring were exposed to elevated temperatures, to a Vibrio challenge or to a combination of the two environmental factors (NC > VC, VH, NH). The expression of calcrul was decreased if offspring were kept at elevated temperatures (NC, VC, VH > NH). *Vibrio* immune‐challenged induced expression of *AIF* and *MYST* to a higher extent than a sole temperature increase or the combination of a temperature increase with the immune challenge (VC > VH, NH > NC). The expression of *C9* was upregulated upon an immune challenge, but not under elevated temperatures; if a combination of immune challenge and elevated temperatures was applied, the expression was intermediate (VC > VH > NC, NH) (Figure 6, Tables S1–S5).

The impact of the two parental environmental conditions interacted with the offspring temperature exposure (tparent × Vparent × toffspring) for expression of *LectpII, Kin, Ik.cyto* (innate immune system), *IgM, Lympcyt* (adaptive), *C1* (complement), *Hemk2, No66* (histone methylation), *BROMO* (histone acetylation). If parents were exposed to a heatwave and injected with *Vibrio*, their offspring kept at hot temperatures (VHH) had lowest expression of *LectpII, Ik.cyto, C1* and *BROMO*. This suggests that the offspring of parents that were kept in hot water and exposed to *Vibrio* had a lower gene expression, when offspring also lived in hot water. If low expression is less costly, this would be adaptive TGP. All combinations with parental *Vibrio* exposure induced expression of *Hemk2* and *Kin*, almost independent of the parental and offspring temperature. The dominating *Vibrio* effect on *Hemk2* and *Kin* expression supports the presence of TGIP. Parental exposure to cold temperatures resulted in a lower offspring *IgM* expression (induced antigen recognition) than an exposure of both parents and offspring to hot temperatures; all combinations with parental immunological activation (*Vibrio* injection) were intermediate. Temperature had a stronger impact on *IgM* expression, as indicated by the highest expression of *IgM* in case of hot temperatures in parental environment in combination with hot temperature in offspring environment. This speaks for an adaptive TGP effect, where matching parental and offspring temperature induced gene expression. Elevated temperature experience during the parental generation downregulated *Lympcyt* expression in offspring, while parental *Vibrio* exposure induced its expression, but only under cold temperatures (NHC [parents: NH, offspring: C], VHH [parents: VH, offspring: H] < VCC [parents: VC, offspring: C], VCH [parents: VC, offspring: H]). If parents were kept at cold temperature and did not experience a *Vibrio* injection, offspring showed lowest expression of *No66* if they were also kept at cold temperature (NCC; Figure 7).

**Figure 8:**
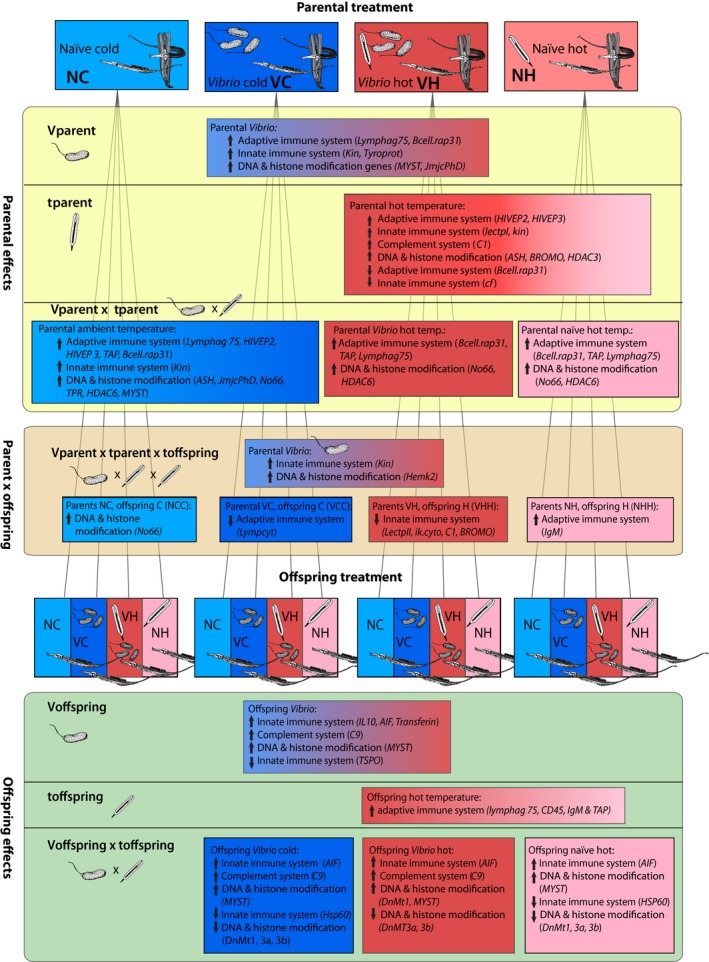
A schematic summary of the experimental design and all results. The parental treatments are displayed in light blue for the naïve cold environment (NC), dark blue for the Vibrio cold environment (VC), red for the Vibrio hot environment (VH) and pink for the naïve hot environment (NH). Panels with yellow background display sole parental effects (Vparent, tparent Vparent × tparent), panels with orange background demonstrate the significant parent × offspring interactions (Vparent × tparent × toffspring), and green panels show the offspring effects (Voffspring, toffspring, Voffspring × toffspring)

In summary, there was only one case for adaptive TGP supported by induced offspring gene expression (*IgM*), indicative for adaptive TGP. Rather we found that a change in two parental environmental conditions (*Vibrio* challenge and temperature alteration) and experience of higher temperatures during offspring development (combination VHH) resulted in a reduced expression of several genes, *LectpII, Ik.cyto, C1, BROMO*, which underlines the presence of TGP. Classical TGIP effects were found for *Kin* and *Hemk2* but revealed only under cold parental temperature a clear induction of gene expression in case of a parental *Vibrio* exposure. The experimental design and all gene expression results are graphically summarized in Figure 8.

### 3.2 Life‐history parameters

Duration of pregnancy was shorter if parents were kept at elevated temperatures, and parental immune challenge did not influence length of pregnancy (ANOVA; Vparents (*df* = 1, *F* = 0.60) *p* = .559, tparents (*df* = 1, *F* = 8.79) *p* < .001, Vparents × tparents (*df* = 1, *F* = 0.46) *p* = .650). Clutch size was neither affected by the parental immune treatment nor by temperature (ANOVA; Vparents (*df* = 1, *F* = 1.14) *p* = .268, tparents (*df* = 1, *F* = 0.09) *p* = .930, Vparents × toffspring (*df* = 1, *F* = 0.87) *p* = .396).

None of the factors assesses had an impact on offspring size that was measured 8 days after birth (linear mixed effect model). This suggests that during the shorter pregnancy at hot temperatures, development must have been accelerated and that the temperature regime juveniles were exposed to after birth did not influence size (Table S6).

## 4 Discussion

Trans‐generational plasticity has the potential to compensate the negative impact of environmental change in sensitive early life stages (Bonduriansky & Day, 2009; Donelson et al., 2012; Kristensen, 1995; Roth, Klein, et al., 2012; Salinas & Munch, 2012; Uller, 2008). Because the resources allocated to plasticity are limited, the adaptive capacity for response to multiple environmental factors may also be constrained (Bubliy, Kristensen, Kellermann, & Loeschcke, 2011). Analogous to negative trait correlations (Etterson & Shaw, 2001; Stearns & Magwene, 2003), a full adjustment to two parental exposures may thus be impossible in the offspring. In this study, heat stress dominated TGP and limited the ability of pipefish offspring to respond to parental immunological challenge.

Consistent with earlier studies in the pipefish *S. typhle,* parental *Vibrio* exposure induced offspring gene expression at a suite of immune genes and genes involved in DNA modification and histone modification supporting TGIP at ambient (18°C [cold]) temperatures (Beemelmanns & Roth, 2016a, 2016b, 2017; Roth, Klein, et al., 2012). A significant induction of expression upon parental *Vibrio* exposure was found for *Kin* (kinesin, involved in intracellular transport), *Tyroprot* (TYRO protein kinase, a cytokine receptor signalling T‐ and B‐cell activation), *Lymphag 75* (lymphocyte antigen 75, involved in antigen presentation) and *Bcell.rap31* (B‐cell activation) associated with the adaptive immune system. Offspring of parents exposed to a bacterial challenge seem to profit from an activated adaptive immune defence. The enhanced expression of *JmjcPhD* (lysine‐specific‐demethylase 5B, histone demethylation) and *MYST* (histone acetyltransferase, histone acetylation) upon parental bacterial exposure indicates a more rigorous gene expression regulation in offspring, as both genes are involved in the control of gene expression over histone modification (Figure 2). If parents were exposed to elevated temperatures (23°C [hot]) in combination with an exposure to *Vibrio*, the impact of TGIP almost disappeared (Figure 5). This supports the hypothesized interactive effects of parental immune challenge and exposure to elevated temperature on offspring gene expression. No synergistic beneficial effects could be identified. We rather find evidence for constrained TGP. It seems that the costly investment into offspring immune defence vanished, when a second environmental challenge was present in the parental generation. Twelve of 44 genes were affected by the interaction of the two environmental factors that were manipulated during the parental generation (Vparent × tparent) (in addition to five of the six genes (not *Tyroprot*) mentioned above with the main Vparent effect: *Hivep2, Hivep3* (human immunodeficiency virus type I enhancer 2 and human immunodeficiency virus type I enhancer 3, involved in VDJ recombination (the process by which T and B cells randomly assemble different gene segments, variable (V), diversity (D) and joining (J) segments, to generate antigen receptors) and MHC binding), *TAP* (TAP‐binding protein tapasin, transport of antigenic peptides) (all three adaptive immune system), *ASH* (histone methyltransferase, gene activation), *No66* (lysine‐specific‐histone demethylase, gene silencing) and *TPR* (lysine‐specific demethylase, gene activation) and *HDAC6* (histone deacetylase, gene silencing) (all four associated with DNA modification and histone modification). The expression of eleven of these twelve genes was highest if parents were exposed to an immunological activation with *Vibrio* bacteria at ambient temperatures (parental VC treatment), implying a boost of offspring immune defence upon parental *Vibrio* challenge (Beemelmanns & Roth, 2016a, 2016b; Roth, Klein, et al., 2012). The expression of several genes (*Bcell.rap31, TAP, Lymphag 75, No66, HDAC6*) was also induced if parents were kept either only at elevated temperatures or in a combination with a *Vibrio* exposure. This implies that the investigated candidate genes react not only to an immune challenge but also to a more general application of stress, that is, an elevation of water temperatures. The parental phenotypic plasticity assigned to handling immunological challenges and temperature stress may thus involve similar physiological mechanisms. However, upon a temperature shift (NH, VH), only the expression of *Lymphag 75* reached the expression level of the sole parental *Vibrio* exposure (VC), and all other genes were only slightly upregulated compared to the control group in which parents were kept at ambient temperatures and did not receive an immunological activation. The application of an immune challenge during the parental generation consequently only had a strong impact at ambient temperatures. A temperature change during the parental generation hampered TGIP and only resulted in a slight upregulation of gene expression, independent of the immune challenge applied. That immune‐challenged pregnant pipefish males actively avoid warm waters (Landis, Sundin, et al. 2012) could potentially underline the importance of TGIP.

Resources that parents invested at cold temperatures into TGIP were at warm temperatures most likely allocated into own metabolism and the accelerated pregnancy. Accordingly, offspring from parents kept at hot temperatures had the same size after a shorter pregnancy (Table S6). While temperature acclimation was most likely not applied early enough to adjust the maternal investment into the eggs, duration of pregnancy was observed to affect size of newborns considerably (O. Roth, unpublished data). Hence, it is tempting to speculate that under elevated temperature, compensatory embryo growth occurred during pregnancy. Offspring from parents that experienced rising temperatures had a lower expression in eight of ten significantly affected genes compared to offspring from parents kept at cold temperatures. Immune genes *LectpI* (lectin protein type I, pathogen recognition receptor), *Kin* (kinesin, intracellular transport) from the innate immune system, *HIVEP 2* and *HIVEP3* from the adaptive immune system and *C1* (recognition subcomponent, formation of the antigen–antibody complex) from the complement system were all downregulated in offspring if parents were exposed to elevated temperatures (Figure 1). This suggests a less efficient immune defence when parents experienced a rise of temperature, which goes in line with our finding of decreased pipefish immune response at warmer temperatures (Landis, Kalbe, et al., 2012). In addition, also *ASH*,* HDAC3* and *BROMO* involved in histone modification were downregulated under these conditions, suggesting a rearrangement (remodelling) of gene expression via histone modification. No effects on DNA methylation were identified. Only *Cf* (coagulation factor II, blood clotting and inflammation) and *Bell.rap31* (B‐cell activation) showed the opposite pattern. This main effect of parental temperature is, however, also influenced by the above‐discussed impact of immune challenge that was only effective at ambient temperatures as indicated by the significant interaction of the two parental effects (Vparent × tparent). Within the limited number of genes addressed in this study, we cannot find support for the hypothesized segregated resource pools. Nevertheless, it is very likely that parental exposure to elevated temperature rather induced a set of genes related to heat stress. With one exception (*Hsp60*), we did not include heat stress genes in our study. Differential gene expression analyses upon full transcriptome comparison should be much more indicative to answering this hypothesis in a future study.

Juveniles kept at hot temperatures induced the expression of genes of the adaptive immune system. If this constitutive upregulation of gene expression at increased temperatures independent of the parental treatment could also be found under natural conditions, it may imply an adaptive reaction towards more virulent pathogens and spreading infectious disease in warmer waters (Harvell et al. 2002).

The absence of significant tparent × toffspring and Vparent × Voffspring interactions implies that the simple case of matching parental and offspring environment did not result in better offspring performance (Burgess & Marshall, 2014). We identified parental effects upon exposure to heat and immune modification, and a subsequent reaction of the offspring towards the two environmental factors applied. In terms of gene expression, the only parent × offspring interaction was identified for the two parental environmental factors with the temperature regime during offspring development (tparent × Vparent × toffspring), while the four‐way interaction was again nonsignificant. This indicates that the combined parental effects upon temperature change and immune challenge depended on the temperature the offspring were kept at, which underlines the presence of TGP. Disentangling the patterns for the nine genes affected, only for *IgM* (immunoglobulin M light chain, pathogen recognition) an upregulation of expression in case of matching parental and offspring environment was detected implying adaptive TGP. However, the downregulation of *LectpII, Ik.cyto, C1, BROMO* in case of matching parental and offspring temperature conditions could also point towards adaptive TGP, as a decreased expression might be more cost‐effective.

Alternatively, the significant interaction among parental temperature exposure, parental immune challenge and the temperature offspring were kept at (tparent × Vparent × toffspring) which may rather imply that TGP reaches its limits once it needs to be attributed to two changing parental environmental factors (Bubliy et al., 2011). This effect was consistently identified for immune genes and DNA‐ and histone‐modification candidate genes investigated in this study. Of nine genes with an impact, four (*LectpII* [lectin protein type II, pathogen recognition receptor], *Ik.cyto* [ik cytokine, inhibits downregulation of MHC], *C1* [recognition subcomponent, forms antigen–antibody complex] and *BROMO* [histone acetyltransferase]) show lowest expression if parents were exposed to a combination of *Vibrio* and elevated temperature, and if hot temperature conditions were met in the offspring environment. While both a parental exposure to an immune challenge in ambient temperature and higher temperatures during juvenile development induced gene expression, the combination of immune challenge with elevated temperature in the parental generation resulted in a downregulation of several genes. This could speak for antagonistic parental effects on immune genes and genes involved in DNA modification and histone modification, if two environmental factors are altered simultaneously that affect the same physiological mechanisms. As chronic stress suppresses immune response (Bonga, 1997), warming environmental conditions could induce susceptibility for parasite infections and disease development.

Recent whole transcriptome gene expression (RNAseq) approaches identified several immune genes involved in thermal TGP (Shama et al., 2016; Veilleux et al., 2015). Immune genes thus seem to reflect the processes of both thermal trans‐generational acclimatization and TGIP. The immune genes investigated in our study are known to correlate with cellular immune defence (Beemelmanns & Roth, 2016b; Birrer et al., 2012), implying their role in host physiology, which adds to the aim to understand the molecular basis of TGP. Epigenetic marks were recently claimed to mediate TGP (Munday, 2014). While studies already confirmed that DNA‐ and histone‐modification genes are influenced by TGIP in pipefish (Beemelmanns & Roth, 2016a, 2016b, 2017), epigenetic regulation also mediates thermal trans‐generational adjustments. *ASH*,* HDAC3* and *BROMO* (acetylation) were downregulated in offspring upon parental heat stress. The downregulation of the histone methyltransferase *ASH* could imply that parental temperature challenge negatively influences embryonic development, yet development was faster at elevated temperature with no negative effect on offspring size. The lower expression of the histone deacetylase *HDAC3* will enhance deacetylation of lysine residues and induce transcription, while downregulation of the histone acetyltransferase *BROMO* will result in negative regulation of transcription over chromatin structure rearrangement.

Benefits of TGP are always context dependent (Marshall, 2008), and TGP can be maladaptive (Schade, Clemmesen, & Wegner, 2014) even if parental and offspring conditions match. While the parental immune challenge induced offspring gene expression, elevated temperature in the parental generation had a smaller impact on the offspring gene expression profiles. The combination of the two parental effects revealed the same pattern as the sole application of a temperature change in the parental generation. We thus identified a dominant parental temperature effect, as the offspring gene expression upon an elevated parental temperature exposure remained. Independent of the applied parental immune challenge, temperature was the master regulator of phenotypic plasticity. Our data suggest that the potential of trans‐generational effects to compensate stressful environmental conditions during offspring maturation is hampered when multiple environmental stressors are applied simultaneously in the parental and offspring generation, potentially because the capacity for TGP is limited. This sheds new light on how animals can cope with changing environmental conditions in nature that usually impact several abiotic and biotic factors simultaneously, and may raise the question whether phenotypic plasticity remains an effective short‐term response that permits acclimation to global change.

## Data archiving statement

All raw data are archived and accessible at PANGAEA (https://www.pangaea.de) under doi:10.1594/PANGAEA.872606.
