# Supplementary material for: Trans‐generational plasticity in response to immune challenge is constrained by heat stress
Source: Evol Appl. 2017 Apr 14;10(5):514–28. doi: 10.1111/eva.12473 (PMC5427669; doi:10.1111/eva.12473)
Supplement: Supplementary file 1 [file EVA-10-514-s001.docx]

**Supplement Tables 1-5:** Linear Mixed Effect Models (LMERs) for all genes assessed, displayed for the functional categories. Supplement Table1: adaptive immune genes (ANOVAs adaptive), Supplement Table 2: innate immune genes (ANOVAs innate), Supplement Table 3: complement component (ANOVAs complement), Supplement Table 4: methylation/ demethylation genes (ANOVAs methylation), Supplement Table 5: acetylation/ deacetylation genes (ANOVAs acetylation). Tparent (tpar), Vparent (Vpar), toffspring (toff) and Voffspring (Voff) were included as fixed factors with all interactions. Family was included as random factor. All factors and interactions that were significant in the functional categories PERMANOVAs are in ***bold*** letters, and only those are indicated with asterisks * in case p< 0.05. Posthoc tests (student t tests) indicate detailed examination of the significant factors and their interactions.

**Supplement Tables 6:** Linear Mixed Effect Model (LMER) for offspring size. Tparent (tpar), Vparent (Vpar), toffspring (toff) and Voffspring (Voff) were included as fixed factors with all interactions. Family was included as random factor.

| Supplement Table1 |  |  |  |  |  |  |  |  |  |  |  |  |  |  |  |  |  |
| --- | --- | --- | --- | --- | --- | --- | --- | --- | --- | --- | --- | --- | --- | --- | --- | --- | --- |
| ANOVAs adaptive |  |  | lymphag 75 | |  | HIVEP 2 | |  | HIVEP3 | |  | CD45 |  |  | Integrin | |  |
| factors | NumDF | DenDF | F.value | Pr (>F) | posthoc | F.value | Pr (>F) | posthoc | F.value | Pr (>F) | posthoc | F.value | Pr(>F) | posthoc | F.value | Pr(>F) | posthoc |
| ***tpar*** | 1 | 276 | 0.305 | **0.591** |  | 5.343 | ***0.042**** | *C>H* | 4.900 | ***0.045**** | *C>H* | 0.108 | **0.748** |  | 0.191 | **0.669** |  |
| ***Vpar*** | 1 | 276 | 4.660 | ***0.046**** | *V>N* | 1.659 | **0.219** |  | 0.036 | **0.851** |  | 2.215 | **0.151** |  | 0.063 | **0.803** |  |
| **toff** | 1 | 276 | 5.803 | ***0.017**** | *H>C* | 0.012 | **0.911** |  | 0.219 | **0.640** |  | 6.377 | ***0.012**** | *H>C* | 0.208 | **0.646** |  |
| Voff | 1 | 276 | 0.002 | 0.966 |  | 1.141 | 0.286 |  | 4.436 | *0.036** | *V=N* | 0.009 | 0.924 |  | 1.413 | 0.346 |  |
| ***tpar:Vpar*** | 1 | 276 | 16.950 | ***0.007**** | *VC>NH>VH>NC* | 5.824 | ***0.031**** | *VC>NC,HN,HV* | 6.588 | ***0.019**** | *VC>NC,NH>VH* | 1.719 | **0.203** |  | 0.859 | **0.363** |  |
| tpar:toff | 1 | 276 | 0.209 | 0.648 |  | 0.294 | 0.588 |  | 0.147 | 0.702 |  | 0.213 | 0.645 |  | 2.496 | 0.115 |  |
| Vpar:toff | 1 | 276 | 0.153 | 0.696 |  | 1.026 | 0.312 |  | 2.722 | 0.100 |  | 1.440 | 0.231 |  | 2.782 | 0.097 |  |
| tpar:Voff | 1 | 276 | 1.506 | 0.221 |  | 0.690 | 0.407 |  | 0.052 | 0.818 |  | 1.399 | 0.238 |  | 0.014 | 0.907 |  |
| Vpar:Voff | 1 | 276 | 0.066 | 0.797 |  | 8.057 | *0.005** |  | 0.428 | 0.514 |  | 0.962 | 0.327 |  | 3.604 | 0.058 |  |
| ***toff:Voff*** | 1 | 276 | 0.036 | **0.846** |  | 2.138 | **0.145** |  | 0.028 | **0.868** |  | 0.237 | **0.626** |  | 2.275 | **0.133** |  |
| ***tpar:Vpar:toff*** | 1 | 276 | 3.536 | **0.061** |  | 2.653 | **0.105** |  | 3.039 | **0.083** |  | 2.171 | **0.142** |  | 0.583 | **0.446** |  |
| tpar:Vpar:Voff | 1 | 276 | 0.078 | 0.781 |  | 3.890 | *0.049** |  | 5.411 | *0.021** |  | 2.823 | 0.094 |  | 1.542 | 0.215 |  |
| tpar:toff:Voff | 1 | 276 | 0.932 | 0.335 |  | 0.618 | 0.432 |  | 1.769 | 0.185 |  | 1.163 | 0.281 |  | 0.577 | 0.448 |  |
| Vpar:toff:Voff | 1 | 276 | 3.033 | 0.083 |  | 3.181 | 0.076 |  | 0.332 | 0.565 |  | 4.290 | *0.039** |  | 2.797 | 0.096 |  |
| tpar:Vpar:toff:Voff | 1 | 276 | 0.159 | 0.690 |  | 0.172 | 0.678 |  | 0.243 | 0.622 |  | 1.108 | 0.294 |  | 0.673 | 0.413 |  |
| std.dev. *family* |  |  | 0.171 |  |  | 0.147 |  |  | 0.227 |  |  | 0.578 |  |  | 0.268 |  |  |
| st.dev. residual |  |  | 0.656 |  |  | 0.664 |  |  | 0.566 |  |  | 1.097 |  |  | 0.504 |  |  |
| ANOVAS adaptive |  |  | IgM |  |  | TAP |  |  | bcell.rap31 | |  | lympcyt | |  |  |  |  |
| factors | NumDF | DenDF | F.value | Pr(>F) | posthoc | F.value | Pr(>F) | posthoc | F.value | Pr(>F) | posthoc | F.value | Pr(>F) | posthoc |  |  |  |
| ***tpar*** | 1 | 276 | 0.355 | **0.563** |  | 0.341 | **0.570** |  | 5.747 | ***0.033**** | *H>C* | 1.137 | **0.306** |  |  |  |  |
| ***Vpar*** | 1 | 276 | 3.943 | **0.059** |  | 2.748 | **0.116** |  | 6.726 | ***0.020**** | *V>N* | 1.104 | **0.304** |  |  |  |  |
| **toff** | 1 | 276 | 5.785 | ***0.017**** | *H>C* | 7.890 | ***0.005**** | *H>C* | 0.602 | **0.439** |  | 0.413 | **0.521** |  |  |  |  |
| Voff | 1 | 276 | 0.406 | 0.524 |  | 0.717 | 0.398 |  | 2.146 | 0.144 |  | 0.114 | 0.736 |  |  |  |  |
| ***tpar:Vpar*** | 1 | 276 | 1.062 | **0.313** |  | 6.221 | ***0.023**** | *VC>NH,VH>NC* | 5.184 | ***0.037**** | *VC,NH,VH>NC* | 1.613 | **0.216** |  |  |  |  |
| tpar:toff | 1 | 276 | 3.258 | 0.072 |  | 0.405 | 0.525 |  | 0.101 | 0.751 |  | 0.380 | 0.538 |  |  |  |  |
| Vpar:toff | 1 | 276 | 1.514 | 0.220 |  | 4.332 | 0.038* |  | 0.190 | 0.663 |  | 3.866 | 0.050 |  |  |  |  |
| tpar:Voff | 1 | 276 | 0.066 | 0.797 |  | 1.337 | 0.249 |  | 0.535 | 0.465 |  | 0.726 | 0.395 |  |  |  |  |
| Vpar:Voff | 1 | 276 | 0.043 | 0.836 |  | 0.071 | 0.790 |  | 0.012 | 0.914 |  | 1.640 | 0.201 |  |  |  |  |
| ***toff:Voff*** | 1 | 276 | 0.006 | **0.941** |  | 0.429 | **0.513** |  | 0.840 | **0.360** |  | 1.034 | **0.313** |  |  |  |  |
| ***tpar:Vpar:toff*** | 1 | 276 | 4.144 | ***0.037**** | *NHH>NCC,NCH,NHC* | 3.466 | **0.064** |  | 0.041 | **0.840** |  | 11.537 | ***<0.001**** | *NHH,VHH>VCC,VCH* |  |  |  |
| tpar:Vpar:Voff | 1 | 276 | 0.752 | 0.387 |  | 0.832 | 0.363 |  | 0.001 | 0.908 |  | 0.216 | 0.642 |  |  |  |  |
| tpar:toff:Voff | 1 | 276 | 0.946 | 0.332 |  | 0.914 | 0.340 |  | 0.104 | 0.294 |  | 0.093 | 0.761 |  |  |  |  |
| Vpar:toff:Voff | 1 | 276 | 0.007 | 0.935 |  | 0.014 | 0.905 |  | 0.068 | 0.795 |  | 2.570 | 0.110 |  |  |  |  |
| tpar:Vpar:toff:Voff | 1 | 276 | 0.313 | 0.576 |  | 0.355 | 0.552 |  | 0.554 | 0.457 |  | 0.902 | 0.343 |  |  |  |  |
| std.dev. *family* |  |  | 0.643 |  |  | 0.236 |  |  | 0.149 |  |  | 0.351 |  |  |  |  |  |
| st.dev. residual |  |  | 0.969 |  |  | 0.778 |  |  | 0.718 |  |  | 0.661 |  |  |  |  |  |

| Supplement Table 2 |  |  |  |  |  |  |  |  |  |  |  |  |  |  |  |  |  |
| --- | --- | --- | --- | --- | --- | --- | --- | --- | --- | --- | --- | --- | --- | --- | --- | --- | --- |
| ANOVAS innate |  |  | lectpII |  |  | lectpI |  |  | cf |  |  | hsp |  |  | ik.cyto |  |  |
| factors | Num DF | Den DF | F.value | Pr (>F) | posthoc | F.value | Pr (>F) | posthoc | F.value | Pr (>F) | posthoc | F.value | Pr (>F) | posthoc | F.value | Pr (>F) | posthoc |
| ***tpar*** | 1 | 276 | 2.326 | **0.153** |  | 5.163 | ***0.042**** | *C>H* | 5.711 | ***0.034**** | *H>C* | 2.219 | **0.161** |  | 4.239 | **0.060** |  |
| ***Vpar*** | 1 | 276 | 0.052 | **0.822** |  | 1.691 | **0.208** |  | 0.253 | **0.622** |  | 0.267 | **0.610** |  | 0.097 | **0.759** |  |
| toff | 1 | 276 | 1.641 | 0.201 |  | 0.489 | 0.485 |  | 2.815 | 0.095 |  | 2.337 | 0.127 |  | 0.388 | *0.534* |  |
| ***Voff*** | 1 | 276 | 0.001 | **0.979** |  | 0.978 | **0.324** |  | 0.705 | **0.402** |  | 1.566 | **0.212** |  | 0.824 | **0.365** |  |
| ***tpar:Vpar*** | 1 | 276 | 0.026 | **0.874** |  | 0.128 | **0.724** |  | 0.218 | **0.646** |  | 0.310 | **0.583** |  | 0.405 | **0.532** |  |
| tpar:toff | 1 | 276 | 1.887 | 0.171 |  | 0.488 | 0.854 |  | 0.760 | 0.382 |  | 0.001 | 0.994 |  | 0.006 | 0.941 |  |
| Vpar:toff | 1 | 276 | 0.001 | 0.991 |  | 2.391 | 0.123 |  | 0.340 | 0.560 |  | 3.759 | 0.054 |  | 0.090 | 0.765 |  |
| tpar:Voff | 1 | 276 | 0.178 | 0.673 |  | 1.294 | 0.257 |  | 0.118 | 0.731 |  | 1.118 | 0.291 |  | 1.068 | 0.302 |  |
| Vpar:Voff | 1 | 276 | 0.002 | 0.961 |  | 0.381 | 0.538 |  | 0.001 | 0.991 |  | 2.856 | 0.092 |  | 1.863 | 0.173 |  |
| ***toff:Voff*** | 1 | 276 | 0.032 | **0.859** |  | 0.057 | **0.811** |  | 0.520 | **0.471** |  | 8.285 | ***0.004**** | *CN,VH>HN,VC* | 0.054 | **0.817** |  |
| ***tpar:Vpar:toff*** | 1 | 276 | 6.816 | ***0.001**** | *all>HVH* | 0.015 | **0.903** |  | 0.062 | **0.804** |  | 0.051 | **0.822** |  | 10.738 | ***0.001**** | *all>HVH* |
| tpar:Vpar:Voff | 1 | 276 | 0.217 | 0.642 |  | 0.710 | 0.400 |  | 1.237 | 0.267 |  | 2.502 | 0.118 |  | 0.558 | 0.456 |  |
| tpar:toff:Voff | 1 | 276 | 0.481 | 0.489 |  | 2.944 | 0.087 |  | 3.404 | 0.066 |  | 1.729 | 0.190 |  | 0.013 | 0.908 |  |
| Vpar:toff:Voff | 1 | 276 | 1.846 | 0.175 |  | 0.021 | 0.884 |  | 0.427 | 0.514 |  | 0.062 | 0.804 |  | 8.790 | 0.003* |  |
| tpar:Vpar:toff:Voff | 1 | 276 | 1.979 | 0.161 |  | 2.715 | 0.101 |  | 0.128 | 0.721 |  | 0.017 | 0.897 |  | 0.528 | 0.468 |  |
| std.dev. *family* |  |  | 0.595 |  |  | 0.886 |  |  | 0.270 |  |  | 0.443 |  |  | 0.156 |  |  |
| st.dev. residual |  |  | 1.019 |  |  | 2.089 |  |  | 0.721 |  |  | 0.706 |  |  | 0.519 |  |  |
| ANOVAS innate |  |  | IL10 |  |  | kin |  |  | nramp | |  | TSPO |  |  | LPS:TNF | |  |
| factors | Num DF | Den DF | F.value | Pr (>F) | posthoc | F.value | Pr (>F) | posthoc | F.value | Pr (>F) | posthoc | F.value | Pr (>F) | posthoc | F.value | Pr (>F) | posthoc |
| ***tpar*** | 1 | 276 | 0.176 | **0.681** |  | 7.826 | ***0.016**** | *C>H* | 0.025 | **0.877** |  | 0.325 | **0.578** |  | 0.887 | **0.363** |  |
| ***Vpar*** | 1 | 276 | 3.870 | **0.063** |  | 9.273 | ***0.008**** | *V>N* | 0.108 | **0.746** |  | 0.794 | **0.382** |  | 0.930 | **0.342** |  |
| toff | 1 | 276 | 0.407 | 0.524 |  | 1.969 | 0.162 |  | 0.429 | 0.513 |  | 2.163 | 0.142 |  | 1.123 | 0.290 |  |
| ***Voff*** | 1 | 276 | 33.305 | ***<0.001**** | *V>N* | 0.144 | **0.704** |  | 0.203 | **0.653** |  | 12.182 | ***<0.001**** | *N>V* | 1.234 | **0.288** |  |
| ***tpar:Vpar*** | 1 | 276 | 0.875 | **0.361** |  | 4.886 | ***0.042**** | *CV>CN,HN,HV* | 0.075 | **0.787** |  | 1.538 | **0.227** |  | 0.001 | **0.975** |  |
| tpar:toff | 1 | 276 | 1.157 | 0.283 |  | 0.285 | 0.594 |  | 0.869 | 0.352 |  | 1.162 | 0.282 |  | 0.169 | 0.682 |  |
| Vpar:toff | 1 | 276 | 0.259 | 0.611 |  | 0.142 | 0.707 |  | 0.001 | 0.990 |  | 4.979 | *0.026** |  | 0.078 | 0.780 |  |
| tpar:Voff | 1 | 276 | 0.338 | 0.562 |  | 1.857 | 0.175 |  | 0.029 | 0.865 |  | 2.480 | 0.116 |  | 0.293 | 0.589 |  |
| Vpar:Voff | 1 | 276 | 0.063 | 0.802 |  | 2.521 | 0.114 |  | 0.205 | 0.651 |  | 1.936 | 0.165 |  | 0.912 | 0.340 |  |
| ***toff:Voff*** | 1 | 276 | 2.233 | **0.136** |  | 0.869 | **0.352** |  | 0.716 | **0.398** |  | 0.718 | **0.398** |  | 0.156 | **0.693** |  |
| ***tpar:Vpar:toff*** | 1 | 276 | 6.099 | ***0.014**** |  | 6.328 | ***0.013**** | *CVC,CVH>all* | 3.800 | **0.052** |  | 0.756 | **0.385** |  | 14.387 | ***<0.001**** |  |
| tpar:Vpar:Voff | 1 | 276 | 0.138 | 0.711 |  | 4.713 | *0.031** |  | 1.801 | 0.181 |  | 1.154 | 0.284 |  | 0.349 | 0.555 |  |
| tpar:toff:Voff | 1 | 276 | 0.040 | 0.842 |  | 0.591 | 0.443 |  | 0.048 | 0.828 |  | 0.769 | 0.381 |  | 1.114 | 0.292 |  |
| Vpar:toff:Voff | 1 | 276 | 1.942 | 0.165 |  | 1.845 | 0.176 |  | 0.177 | 0.675 |  | 0.225 | 0.636 |  | 0.001 | 0.985 |  |
| tpar:Vpar:toff:Voff | 1 | 276 | 0.365 | 0.546 |  | 0.578 | 0.444 |  | 0.385 | 0.556 |  | 1.068 | 0.302 |  | 0.012 | 0.911 |  |
| std.dev. *family* |  |  | 0.721 |  |  | 0.119 |  |  | 0.172 |  |  | 0.183 |  |  | 1.132 |  |  |
| st.dev. residual |  |  | 1.838 |  |  | 0.522 |  |  | 0.447 |  |  | 0.373 |  |  | 1.346 |  |  |

| ANOVAS innate |  |  | calcrul |  |  | intf |  |  | IL8 |  |  | Tyroprot | |  | ck7 |  |  |
| --- | --- | --- | --- | --- | --- | --- | --- | --- | --- | --- | --- | --- | --- | --- | --- | --- | --- |
| factors | Num DF | Den DF | F.value | Pr (>F) | posthoc | F.value | Pr (>F) | posthoc | F.value | Pr (>F) | posthoc | F.value | Pr (>F) | posthoc | F.value | Pr (>F) | posthoc |
| ***tpar*** | 1 | 276 | 1.176 | **0.299** |  | 0.662 | **0.430** |  | 0.617 | **0.447** |  | 8.047 | **0.015*** |  | 0.194 | **0.667** |  |
| ***Vpar*** | 1 | 276 | 0.018 | **0.896** |  | 0.024 | **0.879** |  | 0.396 | **0.533** |  | 20.806 | **<0.001*** | V>N | 2.202 | **0.154** |  |
| toff | 1 | 276 | 8.582 | 0.004* | H=C | 1.500 | 0.222 |  | 0.017 | 0.895 |  | 0.358 | 0.550 |  | 0.105 | 0.747 |  |
| ***Voff*** | 1 | 276 | 1.614 | **0.205** |  | 2.708 | **0.101** |  | 1.977 | **0.161** |  | 0.023 | **0.878** |  | 0.038 | **0.847** |  |
| ***tpar:Vpar*** | 1 | 276 | 0.785 | **0.384** |  | 2.943 | **0.098** |  | 1.552 | **0.222** |  | 3.319 | **0.090** |  | 0.997 | **0.330** |  |
| tpar:toff | 1 | 276 | 0.595 | 0.441 |  | 0.289 | 0.591 |  | 6.598 | 0.011* |  | 0.063 | 0.802 |  | 0.626 | 0.429 |  |
| Vpar:toff | 1 | 276 | 1.105 | 0.294 |  | 5.661 | *0.018** |  | 0.276 | 0.600 |  | 0.108 | 0.742 |  | 0.004 | 0.946 |  |
| tpar:Voff | 1 | 276 | 0.601 | 0.439 |  | 0.096 | 0.756 |  | 0.715 | 0.398 |  | 2.553 | 0.111 |  | 0.001 | 0.997 |  |
| Vpar:Voff | 1 | 276 | 7.630 | *0.006** |  | 0.400 | 0.528 |  | 2.129 | 0.146 |  | 2.892 | 0.090 |  | 4.183 | 0.042 |  |
| ***toff:Voff*** | 1 | 276 | 11.139 | ***<0.001**** | *NC,VC,VH>NH* | 2.524 | **0.113** |  | 0.001 | **0.975** |  | 0.427 | **0.514** |  | 0.688 | **0.408** |  |
| ***tpar:Vpar:toff*** | 1 | 276 | 0.661 | **0.417** |  | 2.327 | **0.128** |  | 0.043 | **0.835** |  | 3.740 | **0.054** |  | 1.627 | **0.203** |  |
| tpar:Vpar:Voff | 1 | 276 | 0.165 | 0.685 |  | 0.146 | 0.702 |  | 0.159 | 0.690 |  | 2.073 | 0.151 |  | 0.018 | 0.984 |  |
| tpar:toff:Voff | 1 | 276 | 0.723 | 0.396 |  | 0.023 | 0.880 |  | 0.016 | 0.900 |  | 0.780 | 0.378 |  | 0.614 | 0.434 |  |
| Vpar:toff:Voff | 1 | 276 | 0.055 | 0.817 |  | 0.013 | 0.910 |  | 0.071 | 0.790 |  | 0.813 | 0.368 |  | 0.985 | 0.322 |  |
| tpar:Vpar:toff:Voff | 1 | 276 | 2.996 | 0.085 |  | 0.060 | 0.807 |  | 3.988 | 0.047* |  | 0.073 | 0.787 |  | 1.241 | 0.266 |  |
| std.dev. *family* |  |  | 0.367 |  |  | 0.503 |  |  | 0.839 |  |  | 0.442 |  |  | 0.553 |  |  |
| st.dev. residual |  |  | 0.594 |  |  | 0.814 |  |  | 1.027 |  |  | 0.555 |  |  | 1.493 |  |  |
| ANOVAS innate |  |  | AIF |  |  | transferin | |  |  |  |  |  |  |  |  |  |  |
| factors | Num DF | Den DF | F.value | Pr (>F) | posthoc | F.value | Pr (>F) | posthoc |  |  |  |  |  |  |  |  |  |
| ***tpar*** | 1 | 276 | 0.467 | **0.506** |  | 0.470 | **0.505** |  |  |  |  |  |  |  |  |  |  |
| ***Vpar*** | 1 | 276 | 0.546 | **0.469** |  | 2.817 | **0.107** |  |  |  |  |  |  |  |  |  |  |
| toff | 1 | 276 | 0.279 | 0.598 |  | 0.145 | 0.703 |  |  |  |  |  |  |  |  |  |  |
| ***Voff*** | 1 | 276 | 19.350 | ***<0.001**** | *V>N* | 8.160 | ***0.005**** | V>N |  |  |  |  |  |  |  |  |  |
| ***tpar:Vpar*** | 1 | 276 | 3.028 | **0.098** |  | 0.954 | **0.339** |  |  |  |  |  |  |  |  |  |  |
| tpar:toff | 1 | 276 | 0.029 | 0.865 |  | 1.683 | 0.196 |  |  |  |  |  |  |  |  |  |  |
| Vpar:toff | 1 | 276 | 1.547 | 0.215 |  | 2.560 | 0.111 |  |  |  |  |  |  |  |  |  |  |
| tpar:Voff | 1 | 276 | 0.453 | 0.501 |  | 0.194 | 0.660 |  |  |  |  |  |  |  |  |  |  |
| Vpar:Voff | 1 | 276 | 0.826 | 0.364 |  | 0.112 | 0.738 |  |  |  |  |  |  |  |  |  |  |
| ***toff:Voff*** | 1 | 276 | 7.715 | ***0.006**** | *VC>VH,NH>NC* | **0.658** | **0.418** |  |  |  |  |  |  |  |  |  |  |
| ***tpar:Vpar:toff*** | 1 | 276 | 1.290 | **0.257** |  | **0.165** | **0.685** |  |  |  |  |  |  |  |  |  |  |
| tpar:Vpar:Voff | 1 | 276 | 0.029 | 0.866 |  | 0.776 | 0.379 |  |  |  |  |  |  |  |  |  |  |
| tpar:toff:Voff | 1 | 276 | 1.672 | 0.197 |  | 0.427 | 0.514 |  |  |  |  |  |  |  |  |  |  |
| Vpar:toff:Voff | 1 | 276 | 0.070 | 0.791 |  | 2.582 | 0.109 |  |  |  |  |  |  |  |  |  |  |
| tpar:Vpar:toff:Voff | 1 | 276 | 0.219 | 0.640 |  | 0.308 | 0.579 |  |  |  |  |  |  |  |  |  |  |
| std.dev. *family* |  |  | 0.385 |  |  | 0.317 |  |  |  |  |  |  |  |  |  |  |  |
| st.dev. residual |  |  | 1.119 |  |  | 0.648 |  |  |  |  |  |  |  |  |  |  |  |

| Supplement Table 3 |  |  |  |  |  |  |  |  |  |  |  |
| --- | --- | --- | --- | --- | --- | --- | --- | --- | --- | --- | --- |
| ANOVAs complement | |  | C1 |  |  | C3 |  |  | C9 |  |  |
| factors | Num DF | Den DF | F.value | Pr (>F) | posthoc | F.value | Pr (>F) | posthoc | F.value | Pr (>F) | posthoc |
| ***tpar*** | 1 | 276 | 8.162 | ***0.014**** | *C>H* | 1.690 | **0.222** |  | 3.461 | **0.095** |  |
| ***Vpar*** | 1 | 276 | 3.301 | ***0.084*** |  | 0.267 | **0.611** |  | 4.018 | **0.065** |  |
| toff | 1 | 276 | 4.411 | *0.037** | *H=C* | 6.409 | *0.012** | *H=C* | 0.221 | 0.639 |  |
| ***Voff*** | 1 | 276 | 0.703 | **0.403** |  | 1.570 | **0.211** |  | 23.109 | ***<0.001**** | *V>N* |
| ***tpar:Vpar*** | 1 | 276 | 2.806 | **0.109** |  | 0.166 | **0.687** |  | 1.470 | **0.245** |  |
| tpar:toff | 1 | 276 | 0.074 | 0.785 |  | 4.922 | *0.027** |  | 0.918 | 0.339 |  |
| Vpar:toff | 1 | 276 | 2.327 | 0.128 |  | 0.052 | 0.820 |  | 0.887 | 0.347 |  |
| tpar:Voff | 1 | 276 | 0.765 | 0.383 |  | 0.047 | 0.829 |  | 0.997 | 0.319 |  |
| Vpar:Voff | 1 | 276 | 0.684 | 0.409 |  | 0.864 | 0.354 |  | 0.038 | 0.847 |  |
| ***toff:Voff*** | 1 | 276 | 0.035 | **0.852** |  | 0.084 | **0.773** |  | 4.168 | ***0.042**** | *VC>VH>NC,NH* |
| ***tpar:Vpar:toff*** | 1 | 276 | 5.292 | ***0.022**** | *NCC> all* | 0.227 | **0.634** |  | 0.235 | **0.629** |  |
| tpar:Vpar:Voff | 1 | 276 | 2.819 | 0.094 |  | 0.032 | 0.859 |  | 0.876 | 0.350 |  |
| tpar:toff:Voff | 1 | 276 | 0.230 | 0.632 |  | 2.808 | 0.095 |  | 0.485 | 0.487 |  |
| Vpar:toff:Voff | 1 | 276 | 1.470 | 0.226 |  | 0.215 | 0.643 |  | 0.183 | 0.669 |  |
| tpar:Vpar:toff:Voff | 1 | 276 | 4.092 | *0.044** |  | 0.076 | 0.783 |  | 1.921 | 0.167 |  |
| std.dev. *family* |  |  | 0.245 |  |  | 0.661 |  |  | 0.308 |  |  |
| st.dev. residual |  |  | 0.576 |  |  | 1.081 |  |  | 1.255 |  |  |

| Supplement Table 4 |  |  |  |  |  |  |  |  |  |  |  |  |  |  |  |  |  |
| --- | --- | --- | --- | --- | --- | --- | --- | --- | --- | --- | --- | --- | --- | --- | --- | --- | --- |
| ANOVAs methylation |  |  | JmjcPhD | |  | No66 |  |  | TPR |  |  | DnMt1 | |  | DnMt3a | |  |
| factors | NumDF | DenDF | F.value | P (>F) | posthoc | F.value | Pr(>F) | posthoc | F.value | Pr(>F) | posthoc | F.value | Pr(>F) | posthoc | F.value | Pr(>F) | posthoc |
| ***tpar*** | 1 | 276 | 2.699 | **0.129** |  | 0.319 | **0.582** |  | 4.748 | **0.050** |  | 0.032 | **0.861** |  | 2.850 | **0.115** |  |
| ***Vpar*** | 1 | 276 | 6.350 | ***0.026**** | *V>N* | 3.682 | **0.072** |  | 1.489 | **0.241** |  | 2.040 | **0.172** |  | 0.528 | **0.477** |  |
| toff | 1 | 276 | 0.928 | 0.585 |  | 3.472 | 0.063 |  | 0.005 | 0.942 |  | 0.010 | 0.921 |  | 4.218 | *0.041** | *H=C* |
| ***Voff*** | 1 | 276 | 1.265 | **0.262** |  | 1-794 | **0.182** |  | 0.041 | **0.840** |  | 1.743 | **0.188** |  | 0.351 | 0.554 |  |
| ***tpar:Vpar*** | 1 | 276 | 10.391 | ***0.007**** | *VC>NC,NH,VH* | 7.065 | ***0.017**** | *VC>NH,VH>NH* | 6.310 | ***0.024**** | *VC>NC,NH,VH* | 0.068 | **0.798** |  | 2.056 | **0.169** |  |
| tpar:toff | 1 | 276 | 0.791 | 0.375 |  | 0.538 | 0.464 |  | 0.152 | 0.697 |  | 0.420 | 0.517 |  | 0.166 | 0.684 |  |
| Vpar:toff | 1 | 276 | 0.428 | 0.514 |  | 3.994 | 0.047* |  | 0.023 | 0.880 |  | 3.534 | 0.061 |  | 2.659 | 0.104 |  |
| tpar:Voff | 1 | 276 | 2.252 | 0.135 |  | 0.011 | 0.916 |  | 2.258 | 0.134 |  | 0.883 | 0.348 |  | 0.570 | 0.451 |  |
| Vpar:Voff | 1 | 276 | 0.262 | 0.609 |  | 0.009 | 0.924 |  | 0.007 | 0.933 |  | 1.138 | 0.287 |  | 0.199 | 0.656 |  |
| ***toff:Voff*** | 1 | 276 | 0.233 | **0.630** |  | 3.531 | **0.061** |  | 0.563 | **0.454** |  | 6.795 | ***0.010**** | *NC,VH>NH,VC* | 4.389 | ***0.037**** | *NC>VC,NH,VH* |
| ***tpar:Vpar:toff*** | 1 | 276 | 3.225 | **0.074** |  | 4.252 | ***0.040**** | *VCH,VCC,NHH>NCC* | 0.705 | **0.402** |  | 1.730 | **0.190** |  | 0.110 | **0.741** |  |
| tpar:Vpar:Voff | 1 | 276 | 2.236 | 0.136 |  | 0.226 | 0.635 |  | 2.671 | 0.103 |  | 0.256 | 0.614 |  | 5.488 | *0.020** |  |
| tpar:toff:Voff | 1 | 276 | 0.007 | 0.932 |  | 0.001 | 0.989 |  | 0.706 | 0.402 |  | 0.501 | 0.480 |  | 1.431 | 0.233 |  |
| Vpar:toff:Voff | 1 | 276 | 0.774 | 0.380 |  | 2.351 | 0.126 |  | 0.582 | 0.446 |  | 0.088 | 0.767 |  | 0.769 | 0.381 |  |
| tpar:Vpar:toff:Voff | 1 | 276 | 0.664 | 0.416 |  | 1.137 | 0.287 |  | 0.566 | 0.453 |  | 0.360 | 0.549 |  | 2.824 | 0.094 |  |
| std.dev. *family* |  |  | 0.103 |  |  | 0.152 |  |  | 0.147 |  |  | 0.136 |  |  | 0.146 |  |  |
| st.dev. residual |  |  | 0.628 |  |  | 0.539 |  |  | 0.616 |  |  | 0.515 |  |  | 0.482 |  |  |
| ANOVAs methylation |  |  | DnMt3b | |  | N6admet | |  | ASH |  |  |  |  |  |  |  |  |
| factors | NumDF | DenDF | F.value | Pr(>F) | posthoc | F.value | Pr(>F) | posthoc | F.value | Pr(>F) | posthoc |  |  |  |  |  |  |
| ***tpar*** | 1 | 276 | 1.992 | **0.182** |  | 1.088 | **0.318** |  | 4.868 | ***0.046**** | *C>H* |  |  |  |  |  |  |
| ***Vpar*** | 1 | 276 | 0.538 | **0.471** |  | 1.325 | **0.262** |  | 1.143 | **0.299** |  |  |  |  |  |  |  |
| toff | 1 | 276 | 2.525 | 0.113 |  | 0.727 | 0.394 |  | 0.151 | 0.698 |  |  |  |  |  |  |  |
| ***Voff*** | 1 | 276 | 0.853 | **0.356** |  | 1.049 | 0.307 |  | 0.136 | **0.723** |  |  |  |  |  |  |  |
| ***tpar:Vpar*** | 1 | 276 | 0.207 | **0.654** |  | 0.007 | **0.932** |  | 6.236 | ***0.022**** | *VC>NC,NH,VH* |  |  |  |  |  |  |
| tpar:toff | 1 | 276 | 0.001 | 0.993 |  | 0.622 | 0.431 |  | 0.009 | 0.925 |  |  |  |  |  |  |  |
| Vpar:toff | 1 | 276 | 0.015 | 0.902 |  | 0.352 | 0.554 |  | 0.117 | 0.732 |  |  |  |  |  |  |  |
| tpar:Voff | 1 | 276 | 0.180 | 0.672 |  | 0.249 | 0.618 |  | 0.838 | 0.361 |  |  |  |  |  |  |  |
| Vpar:Voff | 1 | 276 | 0.141 | 0.708 |  | 1.380 | 0.241 |  | 3.182 | 0.076 |  |  |  |  |  |  |  |
| ***toff:Voff*** | 1 | 276 | 6.862 | ***0.009**** | *NC>VC,VH,NH* | 0.283 | **0.595** |  | 0.013 | **0.911** |  |  |  |  |  |  |  |
| ***tpar:Vpar:toff*** | 1 | 276 | 2.083 | **0.150** |  | 1.143 | **0.286** |  | 0.936 | **0.334** |  |  |  |  |  |  |  |
| tpar:Vpar:Voff | 1 | 276 | 1.051 | 0.306 |  | 2.246 | 0.135 |  | 0.434 | 0.511 |  |  |  |  |  |  |  |
| tpar:toff:Voff | 1 | 276 | 1.137 | 0.287 |  | 0.206 | 0.651 |  | 0.062 | 0.803 |  |  |  |  |  |  |  |
| Vpar:toff:Voff | 1 | 276 | 0.001 | 0.980 |  | 0.114 | 0.736 |  | 5.332 | *0.021** |  |  |  |  |  |  |  |
| tpar:Vpar:toff:Voff | 1 | 276 | 2.587 | 0.109 |  | 2.505 | 0.115 |  | 1.565 | 0.212 |  |  |  |  |  |  |  |
| std.dev. *family* |  |  | 0.223 |  |  | 0.361 |  |  | 0.139 |  |  |  |  |  |  |  |  |
| st.dev. residual |  |  | 0.516 |  |  | 0.630 |  |  | 0.429 |  |  |  |  |  |  |  |  |

| Supplement Table 5 |  |  |  |  |  |  |  |  |  |  |  |  |  |  |  |  |  |
| --- | --- | --- | --- | --- | --- | --- | --- | --- | --- | --- | --- | --- | --- | --- | --- | --- | --- |
| ANOVAs acetylation |  |  | HDAC1 | |  | HDAC3 | |  | HDAC6 | |  | Hemk2 | |  | MYST |  |  |
| factors | NumDF | DenDF | F.value | Pr(>F) | posthoc | F.value | Pr(>F) | posthoc | F.value | Pr(>F) | posthoc | F.value | Pr(>F) | posthoc | F.value | Pr(>F) | posthoc |
| ***tpar*** | 1 | 276 | 1.426 | **0.253** |  | 6.453 | ***0.025**** | *C>H* | 0.790 | **0.390** |  | 0.243 | **0.630** |  | 1.152 | **0.303** |  |
| ***Vpar*** | 1 | 276 | 0.231 | **0.636** |  | 0.109 | **0.744** |  | 2.628 | **0.122** |  | 2.457 | **0.131** |  | 5.461 | ***0.032**** | *V>N* |
| toff | 1 | 276 | 0.087 | 0.768 |  | 0.004 | 0.950 |  | 0.019 | 0.892 |  | 4.586 | 0.033* | H=C | 1.999 | 0.159 |  |
| ***Voff*** | 1 | 276 | 0.011 | **0.918** |  | 0.531 | **0.467** |  | 0.046 | **0.830** |  | 0.040 | **0.842** |  | 4.946 | ***0.027**** | *V>N* |
| ***tpar:Vpar*** | 1 | 276 | 0.579 | **0.455** |  | 3.319 | **0.082** |  | 7.490 | **0.013*** | VC>NH,VH>NC | 0.110 | **0.743** |  | 12.106 | ***0.003**** | VC,NH>HV>NC |
| tpar:toff | 1 | 276 | 1.686 | 0.195 |  | 5.580 | *0.019** |  | 0.233 | 0.630 |  | 0.074 | 0.756 |  | 0.277 | 0.599 |  |
| Vpar:toff | 1 | 276 | 0.021 | 0.886 |  | 0.178 | 0.673 |  | 0.251 | 0.617 |  | 7.896 | *0.005** |  | 3.617 | 0.058 |  |
| tpar:Voff | 1 | 276 | 0.327 | 0.568 |  | 0.445 | 0.506 |  | 0.646 | 0.422 |  | 3.515 | 0.062 |  | 0.001 | 0.987 |  |
| Vpar:Voff | 1 | 276 | 0.192 | 0.662 |  | 1.037 | 0.309 |  | 0.007 | 0.933 |  | 2.221 | 0.137 |  | 0.126 | 0.723 |  |
| ***toff:Voff*** | 1 | 276 | 1.204 | **0.273** |  | 0.547 | **0.460** |  | 2.537 | **0.112** |  | 3.614 | **0.058** |  | 7.839 | ***0.005**** | *VC>VH,NH>NC* |
| ***tpar:Vpar:toff*** | 1 | 276 | 3.196 | **0.075** |  | 0.325 | **0.569** |  | 0.045 | **0.832** |  | 0.368 | **0.544** |  | 3.605 | **0.059** |  |
| tpar:Vpar:Voff | 1 | 276 | 1.843 | 0.176 |  | 1.778 | 0.184 |  | 0.324 | 0.570 |  | 5.393 | *0.021** |  | 0.002 | 0.965 |  |
| tpar:toff:Voff | 1 | 276 | 0.286 | 0.593 |  | 0.638 | 0.425 |  | 1.530 | 0.217 |  | 0.586 | 0.445 |  | 0.458 | 0.499 |  |
| Vpar:toff:Voff | 1 | 276 | 3.634 | 0.058 |  | 2.068 | 0.152 |  | 0.001 | 0.993 |  | 1.853 | 0.175 |  | 0.337 | 0.562 |  |
| tpar:Vpar:toff:Voff | 1 | 276 | 4.898 | *0.028** |  | 1.237 | 0.267 |  | 0.578 | 0.448 |  | 0.069 | 0.793 |  | 0.050 | 0.823 |  |
| std.dev. *family* |  |  | 0.122 |  |  | 0.167 |  |  | 0.172 |  |  | 0.230 |  |  | 0.183 |  |  |
| st.dev. residual |  |  | 0.267 |  |  | 0.342 |  |  | 0.465 |  |  | 0.476 |  |  | 0.714 |  |  |
| ANOVAs acetylation |  |  | BROMO | |  |  |  |  |  |  |  |  |  |  |  |  |  |
| factors | NumDF | DenDF | F.value | Pr(>F) | posthoc |  |  |  |  |  |  |  |  |  |  |  |  |
| ***tpar*** | 1 | 276 | 6.718 | ***0.010**** | *C>H* |  |  |  |  |  |  |  |  |  |  |  |  |
| ***Vpar*** | 1 | 276 | 1.202 | **0.274** |  |  |  |  |  |  |  |  |  |  |  |  |  |
| toff | 1 | 276 | 0.115 | 0.735 |  |  |  |  |  |  |  |  |  |  |  |  |  |
| ***Voff*** | 1 | 276 | 0.181 | **0.671** |  |  |  |  |  |  |  |  |  |  |  |  |  |
| ***tpar:Vpar*** | 1 | 276 | 2.748 | **0.099** |  |  |  |  |  |  |  |  |  |  |  |  |  |
| tpar:toff | 1 | 276 | 0.449 | 0.503 |  |  |  |  |  |  |  |  |  |  |  |  |  |
| Vpar:toff | 1 | 276 | 0.019 | 0.891 |  |  |  |  |  |  |  |  |  |  |  |  |  |
| tpar:Voff | 1 | 276 | 0.001 | 0.987 |  |  |  |  |  |  |  |  |  |  |  |  |  |
| Vpar:Voff | 1 | 276 | 0.595 | 0.441 |  |  |  |  |  |  |  |  |  |  |  |  |  |
| ***toff:Voff*** | 1 | 276 | 3.774 | **0.053** |  |  |  |  |  |  |  |  |  |  |  |  |  |
| ***tpar:Vpar:toff*** | 1 | 276 | 5.642 | ***0.018**** | *VCH,VCC,NCC>VHH,NHC,NCH* |  |  |  |  |  |  |  |  |  |  |  |  |
| tpar:Vpar:Voff | 1 | 276 | 0.836 | 0.362 |  |  |  |  |  |  |  |  |  |  |  |  |  |
| tpar:toff:Voff | 1 | 276 | 0.059 | 0.809 |  |  |  |  |  |  |  |  |  |  |  |  |  |
| Vpar:toff:Voff | 1 | 276 | 4.025 | *0.045** |  |  |  |  |  |  |  |  |  |  |  |  |  |
| tpar:Vpar:toff:Voff | 1 | 276 | 0.200 | 0.655 |  |  |  |  |  |  |  |  |  |  |  |  |  |
| std.dev. *family* |  |  | 0.001 |  |  |  |  |  |  |  |  |  |  |  |  |  |  |
| st.dev. residual |  |  | 0.001 |  |  |  |  |  |  |  |  |  |  |  |  |  |  |

| Supplement Table 6 |  |  |  |  |
| --- | --- | --- | --- | --- |
| ANOVAs size |  |  | Size |  |
| factors | NumDF | DenDF | F.value | Pr(>F) |
| ***tpar*** | 1 | 276 | 0.001 | 0.974 |
| Vpar | 1 | 276 | 0.059 | 0.809 |
| ***toff*** | 1 | 276 | 0.068 | 0.794 |
| Voff | 1 | 276 | 1.584 | 0.209 |
| tpar:Vpar | 1 | 276 | 0.001 | 0.990 |
| tpar:toff | 1 | 276 | 1.123 | 0.290 |
| ***Vpar:toff*** | 1 | 276 | 1.827 | 0.178 |
| tpar:Voff | 1 | 276 | 0.071 | 0.791 |
| Vpar:Voff | 1 | 276 | 0.593 | 0.442 |
| toff:Voff | 1 | 276 | 0.788 | 0.376 |
| tpar:Vpar:toff | 1 | 276 | 2.937 | 0.088 |
| tpar:Vpar:Voff | 1 | 276 | 0.448 | 0.504 |
| tpar:toff:Voff | 1 | 276 | 0.872 | 0.351 |
| Vpar:toff:Voff | 1 | 276 | 0.679 | 0.411 |
| tpar:Vpar:toff:Voff | 1 | 276 | 1.091 | 0.297 |
| std.dev. *family* |  |  | 0.111 |  |
| st.dev. residual |  |  | 0.374 |  |
